# Time-varying effects of socio-demographic and economic factors on the use of institutional long-term care before dementia-related death: A Finnish register-based study

**DOI:** 10.1371/journal.pone.0199551

**Published:** 2018-06-21

**Authors:** Kaarina Korhonen, Elina Einiö, Taina Leinonen, Lasse Tarkiainen, Pekka Martikainen

**Affiliations:** 1 Population Research Unit, Faculty of Social Sciences, University of Helsinki, Helsinki, Finland; 2 Department of Social Policy, London School of Economics and Political Science, London, United Kingdom; 3 Max Planck Institute for Demographic Research, Rostock, Germany; 4 Finnish Institute of Occupational Health, Helsinki, Finland; 5 Department of Public Health Sciences, Stockholm University, Stockholm, Sweden; University of West London, UNITED KINGDOM

## Abstract

**Objectives:**

The effects of socio-demographic and economic factors on institutional long-term care (LTC) among people with dementia remain unclear. Inconsistent findings may relate to time-varying effects of these factors as dementia progresses. To clarify the question, we estimated institutional LTC trajectories by age, marital status and household income in the eight years preceding dementia-related and non-dementia-related deaths.

**Methods:**

We assessed a population-representative sample of Finnish men and women for institutional LTC over an eight-year period before death. Deaths related to dementia and all other causes at the age of 70+ in 2001–2007 were identified from the Death Register. Dates in institutional LTC were obtained from national care registers. We calculated the average and time-varying marginal effects of age, marital status and household income on the estimated probability of institutional LTC use, employing repeated-measures logistic regression models with generalised estimating equations (GEE).

**Results:**

The effects of age, marital status and household income on institutional LTC varied across the time before death, and the patterns differed between dementia-related and non-dementia-related deaths. Among people who died of dementia, being of older age, non-married and having a lower income predicted a higher probability of institutional LTC only until three to four years before death, after which the differences diminished or disappeared. Among women in particular, the probability of institutional LTC was nearly equal across age, marital status and income groups in the last year before dementia-related death. Among those who died from non-dementia-related causes, in contrast, the differences widened until death.

**Conclusions:**

We show that individuals with dementia require intensive professional care at the end of life, regardless of their socio-demographic or economic resources. The results imply that the potential for extending community living for people with dementia is likely to be difficult through modification of their socio-demographic and economic environments.

## Introduction

Among the many concerns relating to population ageing is the increasing demand for institutional long-term care (LTC) [1]. In particular, a growing need for dementia-related LTC can be foreseen as rising numbers of people are affected by dementia [2,3]. Progressive dementia is characterised by a non-reversible decline in memory, thinking and behaviour, eventually impairing independence in daily activities. Dementia is, therefore, one of the strongest determinants of admission to institutional LTC [4,5]. Furthermore, periods in institutional care at the end of life are longer for people who die of dementia compared to people who die of any other cause [6]. Because institutional dementia care imposes a heavy economic burden on social and health care sectors, national long-term targets have been to promote community living as an alternative to institutionalisation [7]. Additionally, people themselves commonly prefer to live at home in familiar surroundings where autonomy is easier to maintain. To support independent living and, at the same time, ensure timely access to institutional LTC for those in need, a more comprehensive understanding of the factors contributing to the use of LTC with dementia is needed.

In general, important social and economic resources facilitate living at home through, for example, emotional and instrumental support provided by the spouse, and better access to privately provided home-care services and home ownership. Such resources are likely to be increasingly available to the ageing baby boomers because of the delayed widowhood and improved standard of living. However, although socio-demographic and economic factors are well-established determinants of institutional LTC in the general older population [8–10], the relevance of these factors for the care use of people with dementia remains unknown. On the one hand, social and economic resources may be particularly important to people with dementia, who are highly dependent on informal care. On the other hand, given the high burden on the informal caregiver [11] and the level of cognitive and functional impairment, it is possible that these factors cannot offset the need for intensive professional care at the end of life. Previous studies of people with dementia, which have mainly investigated time to admission to institutional LTC, have offered support for both of these hypotheses; some studies report that older people [11–16], the widowed, and the never married [16,17] have a higher risk of institutionalisation, whereas other studies [12,16,18] including a systematic review [19] found no consistent effects of age, marital status or living arrangements.

A major limitation of most previous research is that the association between LTC and socio-demographic and economic factors has been assumed to be constant across dementia progression. It is feasible, however, that the extent to which one benefits from social and economic resources alters as dementia progresses. Thus, the incompatibility in earlier results may relate to time-varying effects of socio-demographic and economic factors, inconsistently captured in studies estimating time to institutionalisation with different follow-up lengths.

The overall aim of our study was to provide novel evidence on how the effects of socio-demographic and economic factors on institutional LTC change towards the end of life among people with and without dementia. To overcome the limitations of earlier studies, we estimated the associations according to proximity to death, and thus were able to observe potential time-varying effects of these factors. We selected age, marital status and household income as indicators of socio-demographic and economic resources due to their recognised contribution to the care use of the general older population but their unclear influence on people with dementia. Using a population-representative sample linked with the Death Register and national care records, we estimated trajectories of institutional LTC over an eight-year period before death. To permit comparisons to the general older population, we distinguished between dementia-related and all other deaths, and also included those who did not die by the same age as a reference group. Thus, our specific aims were: (i) to estimate the age-adjusted and fully-adjusted associations between institutional LTC and age, marital status and household income, averaged over the eight-year period before dementia-related and non-dementia-related deaths; (ii) to assess how the associations vary according to proximity to death; and (iii) to compare these associations between men and women.

## Methods

### Ethics statement

The study has been approved by Statistics Finland Board of Ethics (permit TK-53-339-13). The data were collected for routine administrative registration purposes and, therefore, informed consent of the participants was not obtained. These register data can be used for scientific purposes under the Personal Data Act and the Statistics Act. Statistics Finland anonymised the data prior to providing them to researchers.

### Data

We used longitudinal register data on the Finnish population, which Statistics Finland has compiled by linking continuously updated population, employment and mortality registers using unique personal identification numbers assigned to all permanent residents. In compliance with the data-protection regulations, Statistics Finland does not release individual-level data on the total population for research purposes. Therefore, an 11% random sample of the total population in 1987–2007 was drawn from the population register. Because regulations concerning deceased individuals are less strict, we obtained an 80% sample of all deaths in this period. To account for the differing sampling probabilities of individuals, all analyses used weights provided by Statistics Finland; lower weight was given to persons who died and higher weight to persons who survived.

### Variables

We assessed the use of institutional LTC in the eight years (2,920 days) preceding death. Deaths attributable to dementia and all other causes at the age of 70 years and above in 2001–2007 were identified from the Death Register of Statistics Finland. Dementia-related deaths included deaths with the International Classification of Diseases 10^th^ revision (ICD-10) codes F00–03 or G30 as the underlying or a contributory cause on the death certificate. Individuals who survived to 31 December 2007 comprised a survivor group for reference, for whom the end of the study period (equivalent to the date of death) was set at December 31 of a randomly assigned year in the period of 2001–2007. The analyses excluded individuals for whom data on household income was unavailable (n = 2,550). Altogether, the study population comprised 248,078 individuals, of whom 36,971 died from dementia, 150,686 died from other causes and 60,421 survived to the end of the study period.

We obtained dates in institutional care in 1995–2007 from institutional discharge registers and patient censuses collected by the National Institute for Health and Welfare. We defined institutional LTC as care periods in nursing homes, service homes with 24-hour assistance, health centres, rehabilitation care, and psychiatric care, when they lasted for at least 90 days or the local authority had granted a long-term care resolution. Based on the continuous time-scale measurement of institutional LTC, we created an annual dummy variable indicating whether the individual had been in institutional LTC in each of the eight 365-day periods dating back from their death or the end of the study period. Thus, the study included people who never entered institutional LTC, entered at some point during the eight years before death, and people who were already in institutional LTC in the eighth year before their death.

The main explanatory variables were age, marital status and household income, indicating the socio-demographic and economic resources available to the person. We used 5-year age groups, categorised as 70–74, 75–79, 80–84, 85–89 and 90+ years, based on age at death (or end of the study period for the survivors). Given the high probability of becoming widowed at these ages, we used marital status as a time-dependent variable, the status being updated at the end of each calendar year. The variable was categorised as married, divorced, widowed and never married. Household income included the household members’ total income subject to state taxation, information about which was obtained from the Finnish Tax Administration and the Social Insurance Institution of Finland. To permit comparisons between different household structures, we divided total household income by the number of consumption units according to the OECD-modified equivalence scale [20]. To avoid institutional residence from affecting household income, we measured income from the year preceding the eighth year before death. In cases where the individual was already in institutional LTC eight years before death, we tracked income from up to four previous years. We used income as a categorical variable divided into quintiles within each birth cohort.

To investigate time-varying associations between institutional LTC and the main explanatory variables, the models included interaction with the proximity to death. Proximity to death indicated the number of years to death (or end of the study period). Linear, squared and cubed terms of proximity to death were used in the regression models. Control variables included calendar year and region of residence based on counties (N = 20) to account for possible temporal and regional variance in the provision of institutional care.

### Statistical analyses

To describe our study population, we calculated unadjusted annual probabilities of institutional LTC, averaged over the eight years preceding death (or end of the study period for the survivors) by gender, cause of death, and the main explanatory variables (age, marital status and household income). We further estimated the age-adjusted probabilities of institutional LTC according to proximity to death by gender and cause of death.

Aiming to analyse the socio-demographic differences in more detail, we estimated two regression models for the associations between institutional LTC and age, marital status and household income, averaged over the eight-year period before death. In model 1, we initially analysed the associations adjusting for age, calendar year and region of residence. Model 2 included all variables, mutually adjusting for age, marital status and household income. To allow the associations between institutional LTC and the explanatory variables to vary between genders, we included gender interaction in each model. Separate models were estimated for each of the cause-of-death groups. Models assessing the effects of age included age in 5-year groups, and when only adjusting for age effects, continuous age and age-squared were used.

Interaction with time was then included in the full model to estimate the time-varying associations between institutional LTC and age, marital status and household income. In these models, we allowed the association between institutional LTC and each explanatory variable to vary by gender and in relation to proximity to death, while adjusting for the main effects of the other variables.

The analyses employed repeated-measures logistic regression models with generalised estimating equations (GEE). The models adopted an autoregressive correlation structure to account for the interdependence of the repeated measures within the subjects, assuming a stronger correlation for observations that were temporally more proximate to each other [21]. All results of these models are presented as estimated probabilities (%) or marginal effects, in other words, the absolute differences in the estimated probability of institutional LTC, and their 95% confidence intervals. To account for the differences in age distribution between the cause-of-death groups, we estimated all associations at the mean age of the total sample (79 years). For all other covariates, the observed values were used [22]. Analyses were conducted using Stata 14 [23], and the analytic code is available in S1 Appendix.

## Results

Table 1 presents the distribution of the study population and the unadjusted annual probabilities of institutional LTC, averaged over the eight years preceding death (or end of the study period). As expected, the probability of institutional LTC was substantially higher among men and women who died from dementia as compared to those who died from other causes. However, regardless of the cause of death, the probability of institutional LTC was related to socio-demographic and economic factors. In general, the probability was higher for older people and the non-married, and somewhat higher for those with a lower household income.

**Table 1:** Distribution^a^ of the study population and unadjusted average annual probability (%) of institutional long-term care (LTC) in the eight-year period before death (or end of the study period for survivors) by age, marital status and household income and by cause of death; Finnish men and women, 1995–2007.

	Cause of death								
	Dementia			Non-dementia			Survived		
	%	% in LTC		%	% in LTC		%	% in LTC	
		Men	Women		Men	Women		Men	Women
Age at death^b^									
70–74	4.7	25.2	38.0	16.9	5.4	7.4	35.7	0.9	1.0
75–79	13.5	27.3	38.2	22.5	6.8	10.7	30.9	1.7	2.0
80–84	24.0	28.2	41.7	24.2	9.1	13.8	20.5	2.9	4.8
85–89	28.8	32.8	46.0	20.2	12.3	18.8	9.3	7.1	9.3
90–	29.0	38.2	52.6	16.2	18.3	27.2	3.6	11.0	17.2
Marital status^c^^,^ ^d^									
Married	31.0	24.5	31.8	40.7	6.1	8.5	54.3	1.3	1.5
Divorced	7.2	38.1	47.4	8.4	11.3	16.3	8.9	3.8	3.9
Widowed	51.1	43.5	49.3	39.9	15.4	19.3	27.7	5.7	5.8
Never married	10.7	38.1	50.4	11.0	12.6	19.9	9.0	4.5	5.5
Household income quintile									
Highest	20.3	29.2	46.0	18.9	8.3	15.7	20.5	1.7	3.3
2nd	22.2	29.8	46.3	21.4	8.6	15.7	21.5	1.6	3.2
3rd	22.3	32.2	46.2	22.0	8.9	16.5	21.7	2.1	3.7
4th	21.4	32.2	46.6	22.0	8.9	17.1	20.5	2.6	4.1
Lowest	13.8	33.7	45.7	15.7	11.2	17.8	15.8	3.5	4.7
Total	100.0	31.0	46.2	100.0	9.0	16.6	100.0	2.1	3.8
N	36,971	10,873	24,425	150,686	66,495	82,263	60,421	22,452	37,756

^a^ Weighted distribution

^b^ Age at the end of the study period for survivors

^c^ Distribution of person-years by time-dependent marital status

^d^ Probability of institutional LTC according to marital status one year before death/end of the study period

**Fig 1:**
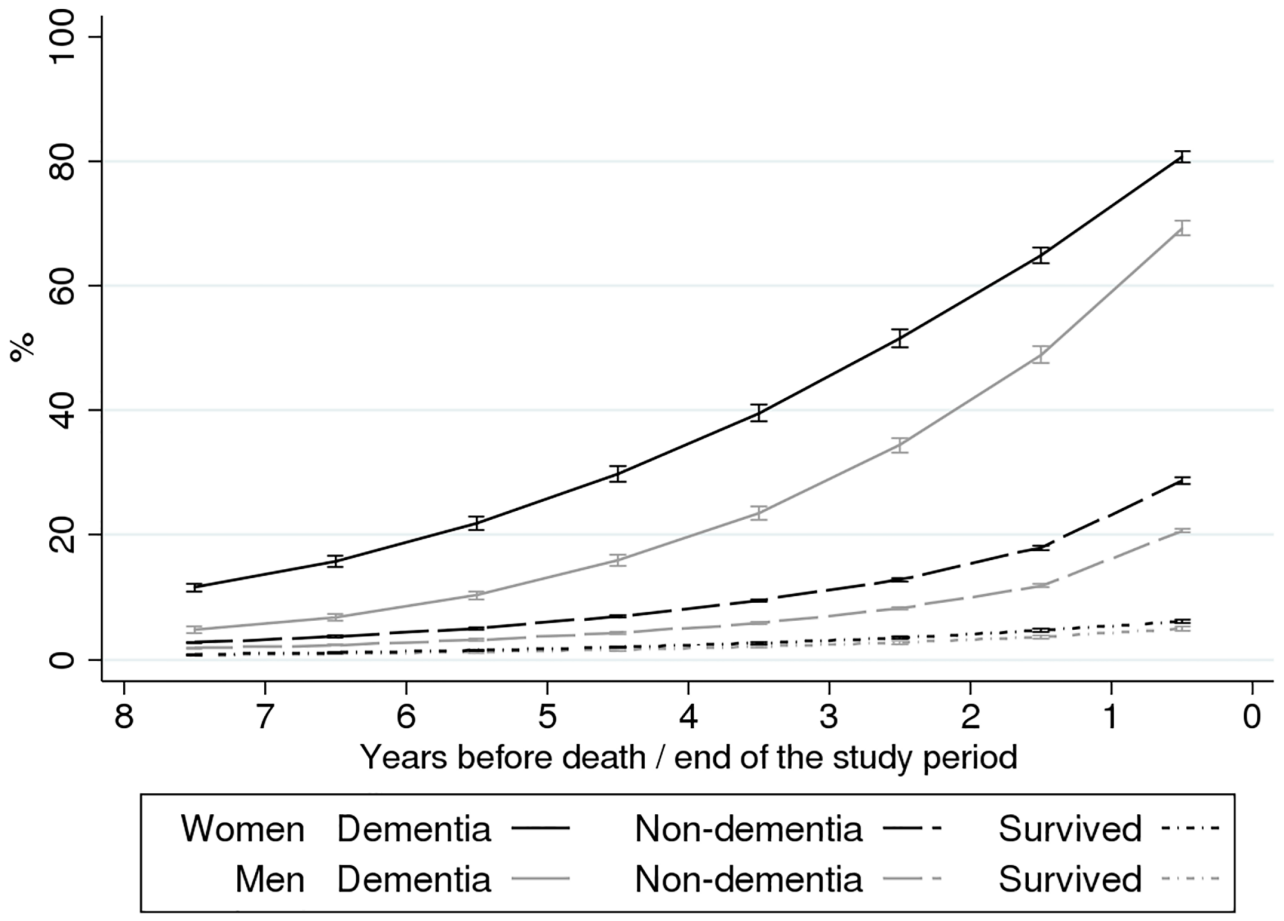
Age-adjusted probability (%) of institutional long-term care (LTC) according to proximity to death (or end of the study period for survivors) by gender and cause of death; Finnish men and women, 1995–2007.

The age-adjusted probability of institutional LTC increased throughout the eight-year period prior to death in all cause-of-death groups (Fig 1). Among people who died from dementia, the probability of LTC increased from 4.8% to 69.2% for men and from 11.6% to 80.7% for women. Among those who died from other causes, the probability of institutional LTC peaked in the last year before death at 20.7% and 28.7% for men and women, respectively. The increase in the probability of LTC appeared to be more concentrated within the final year of life among those who died from causes other than dementia, while among persons who died from dementia, the large increase was more evenly distributed over all eight years preceding death. The likelihood of LTC did not rise above 6.1% during the follow-up among men and women who survived throughout the study period.

### Eight-year average differences in institutional LTC by socio-demographic and economic factors

Average differences in the predicted probability of institutional LTC over the eight-year period are presented in Table 2 for men and Table 3 for women. The estimates were adjusted for age, calendar year and region of residence in model 1, and for all the covariates in model 2. The youngest age group (70–74 years), the married and the highest income quintile were set as the reference groups. In absolute terms, the differences between the age groups 70–74 and 90+ were of a similar magnitude across the three cause-of-death groups among men (10.6–14.0 percentage points; model 1). Among women, in contrast, the age differences were smaller among those who died from dementia (13.3 percentage points, 95% CI 11.3–15.4) than among those who died from other causes (20.5 percentage points, 19.9–21.1). Adjustment for marital status and household income somewhat attenuated the age differences among women, but not men (model 2).

**Table 2:** Average annual differences (Δ) in the probability (%) of institutional long-term care (LTC) and 95% confidence intervals (CI) in the eight-year period before death (or end of the study period for survivors) by age, marital status and household income and by cause of death; Finnish men, 1995–2007.

	Cause of death											
	Dementia				Non-dementia				Survived			
	Model 1		Model 2		Model 1		Model 2		Model 1		Model 2	
	Δ	(95% CI)	Δ	(95% CI)	Δ	(95% CI)	Δ	(95% CI)	Δ	(95% CI)	Δ	(95% CI)
Men												
Age at death^a^												
70–74 (ref.)												
75–79	2.1	(0.1–4.1)	2.7	(0.6–4.8)	1.6	(1.3–2.0)	2.1	(1.7–2.5)	0.9	(0.6–1.1)	1.0	(0.7–1.3)
80–84	3.8	(1.9–5.7)	4.3	(2.3–6.4)	4.2	(3.7–4.6)	5.2	(4.7–5.7)	2.2	(1.8–2.6)	2.6	(2.1–3.1)
85–89	6.8	(4.9–8.8)	6.8	(4.7–8.9)	7.8	(7.3–8.3)	9.0	(8.4–9.5)	6.5	(5.5–7.4)	7.4	(6.3–8.5)
90+	12.2	(10.0–14.3)	10.5	(8.3–12.7)	14.0	(13.3–14.7)	14.4	(13.6–15.1)	10.6	(8.6–12.6)	11.4	(9.2–13.5)
Marital status												
Married (ref.)												
Divorced	13.2	(11.0–15.3)	12.9	(10.8–15.1)	6.7	(6.0–7.4)	6.3	(5.6–6.9)	3.4	(2.5–4.3)	3.3	(2.4–4.2)
Widowed	15.3	(14.2–16.3)	15.2	(14.1–16.3)	5.3	(5.0–5.7)	5.3	(5.0–5.6)	1.6	(1.3–2.0)	1.7	(1.3–2.1)
Never married	13.2	(11.1–15.2)	12.8	(10.7–14.9)	7.4	(6.8–8.0)	6.5	(5.9–7.1)	4.5	(3.5–5.4)	3.8	(2.9–4.7)
Household income quintile												
Highest (ref.)												
2nd	1.0	(-0.3–2.4)	0.9	(-0.6–2.3)	0.8	(0.5–1.2)	0.9	(0.4–1.3)	0.2	(-0.1–0.5)	0.2	(-0.2–0.6)
3rd	4.3	(2.9–5.7)	3.6	(2.1–5.0)	1.5	(1.2–1.9)	1.4	(1.0–1.9)	0.9	(0.5–1.3)	0.9	(0.5–1.4)
4th	5.3	(3.7–6.8)	3.3	(1.7–4.9)	2.2	(1.8–2.6)	1.4	(1.0–1.9)	1.4	(0.9–1.8)	1.2	(0.7–1.7)
Lowest	9.0	(6.9–11.1)	3.4	(1.3–5.5)	6.7	(6.0–7.3)	4.1	(3.5–4.8)	3.6	(2.8–4.5)	2.7	(2.0–3.5)

^a^Age at the end of the study period for survivors

Model 1: each explanatory variable adjusted for age, calendar year and region of residence (estimates for age groups are from a model that includes only the age variable, calendar year and region of residence); Model 2: all variables in the model

**Table 3:** Average annual differences (Δ) in the probability (%) of institutional long-term care (LTC) and 95% confidence intervals (CI) in the eight-year period before death (or end of the study period for survivors) by age, marital status and household income and by cause of death; Finnish women, 1995–2007.

	Cause of death											
	Dementia				Non-dementia				Survived			
	Model 1		Model 2		Model 1		Model 2		Model 1		Model 2	
	Δ	(95% CI)	Δ	(95% CI)	Δ	(95% CI)	Δ	(95% CI)	Δ	(95% CI)	Δ	(95% CI)
Women												
Age at death^a^												
70–74 (ref.)												
75–79	0.7	(-1.5–2.9)	-0.6	(-2.8–1.6)	3.5	(3.0–4.0)	3.1	(2.6–3.6)	1.1	(0.9–1.4)	1.0	(0.8–1.2)
80–84	3.8	(1.7–5.9)	0.7	(-1.3–2.8)	6.9	(6.4–7.4)	5.8	(5.3–6.3)	4.2	(3.8–4.6)	3.6	(3.2–3.9)
85–89	7.4	(5.3–9.5)	2.8	(0.7–4.9)	12.1	(11.6–12.7)	10.0	(9.5–10.5)	8.7	(8.0–9.4)	7.1	(6.5–7.7)
90+	13.3	(11.3–15.4)	7.6	(5.5–9.7)	20.5	(19.9–21.1)	17.1	(16.5–17.7)	16.6	(15.4–17.9)	13.3	(12.2–14.5)
Marital status												
Married (ref.)												
Divorced	12.7	(11.2–14.2)	12.4	(10.9–13.9)	6.2	(5.6–6.8)	5.4	(4.8–6.0)	2.0	(1.5–2.5)	1.6	(1.1–2.0)
Widowed	13.4	(12.6–14.2)	13.3	(12.5–14.1)	5.5	(5.2–5.8)	5.1	(4.8–5.4)	1.5	(1.3–1.7)	1.4	(1.2–1.6)
Never married	14.3	(13.0–15.5)	14.1	(12.8–15.4)	6.9	(6.4–7.5)	6.4	(5.9–6.9)	2.3	(1.8–2.8)	2.0	(1.6–2.5)
Household income quintile												
Highest (ref.)												
2nd	1.2	(0.0–2.3)	1.1	(-0.1–2.2)	0.7	(0.3–1.2)	0.6	(0.2–1.0)	0.3	(0.0–0.6)	0.2	(-0.1–0.5)
3rd	1.9	(0.7–3.0)	1.2	(0.0–2.3)	1.5	(1.1–1.9)	1.1	(0.7–1.5)	0.8	(0.4–1.1)	0.6	(0.3–0.9)
4th	2.4	(1.2–3.5)	0.9	(-0.3–2.0)	2.2	(1.7–2.6)	1.4	(1.0–1.8)	1.1	(0.8–1.5)	0.7	(0.4–1.1)
Lowest	3.9	(2.7–5.2)	1.2	(-0.1–2.4)	4.7	(4.2–5.2)	3.0	(2.6–3.5)	2.1	(1.7–2.5)	1.4	(1.0–1.7)

^a^Age at the end of the study period for survivors

Model 1: each explanatory variable adjusted for age, calendar year and region of residence (estimates for age groups are from a model that includes only the age variable, calendar year and region of residence); Model 2: all variables in the model

Being divorced or widowed or never having been married as opposed to being married were more strongly associated with institutional LTC among men and women who died from dementia (12.7–15.3 percentage points) than among those who died from other causes (5.3–7.4 percentage points) or survived to the end of the study period (1.5–4.5 percentage points; model 1). These differences did not relate to differences in household income (model 2).

The income differences were small in all cause-of-death groups (model 1), being somewhat greater among men (3.6–9.0 percentage points) than among women (2.1–4.7 percentage points) between the highest and lowest income quintiles. Adjusting for marital status further attenuated the differences (model 2).

### Proximity to death

**Fig 2:**
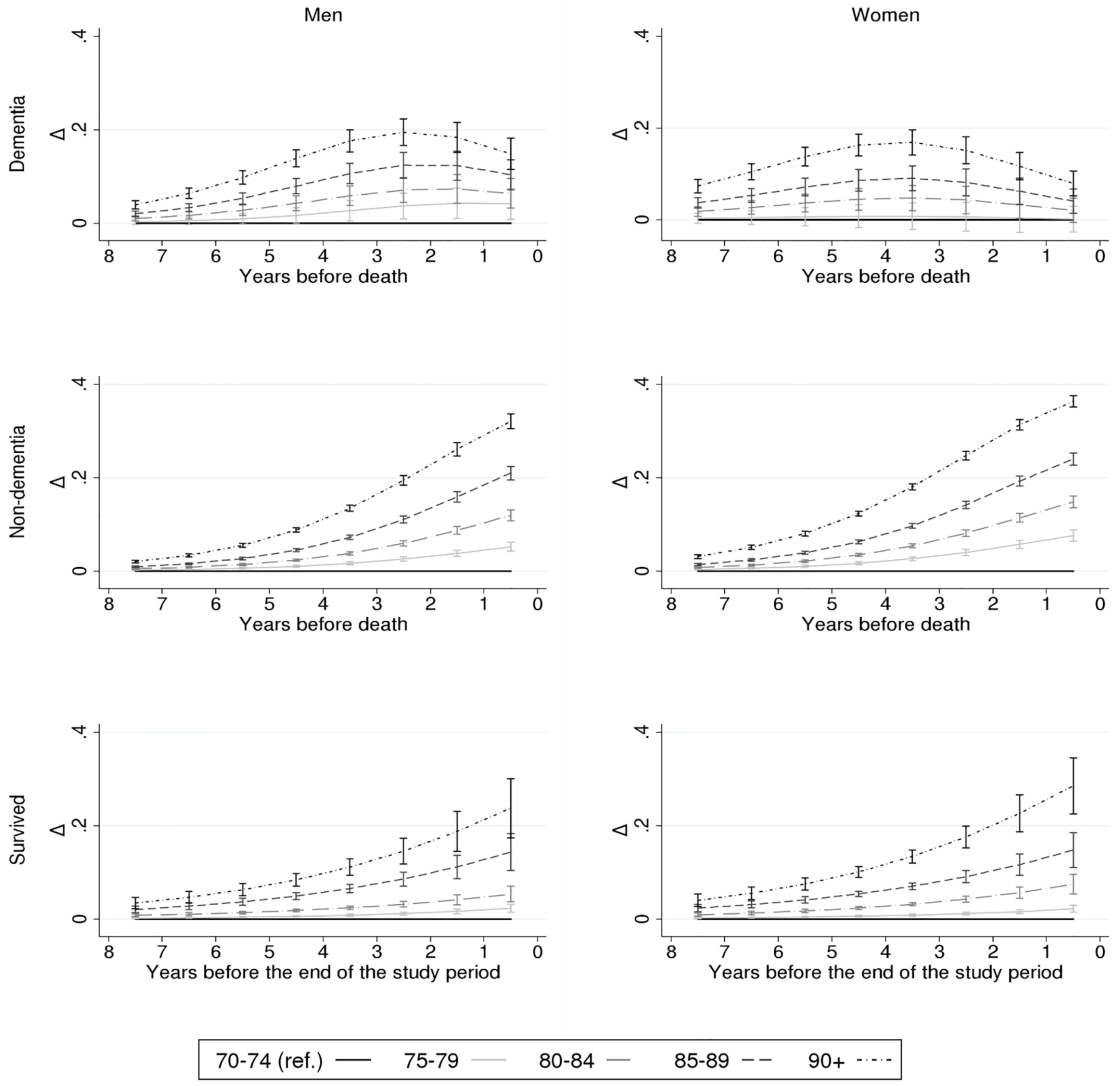
Age differences (Δ) in the probability (%) of institutional long-term care (LTC) according to proximity to death (or end of the study period for survivors) and by gender and cause of death; Finnish men and women, 1995–2007. Adjusted for marital status, household income, calendar year, and region of residence. Please note that the scale on the y axis differs from Figs 3 and 4.

At the next stage, we introduced proximity to death into the full model, and observed that the differences in institutional LTC by age, marital status and household income were not constant over time. In cases of dementia-related death, the differences between age groups increased until 3–4 years before death, where the difference was 16.9–19.4 percentage points between the youngest and the oldest groups (Fig 2). After this peak, the differences substantially reduced among men, and nearly disappeared among women. Among those who died from non-dementia-related causes and among the survivors, the differences between the youngest and oldest age groups continued to increase almost linearly until the last year before death.

**Fig 3:**
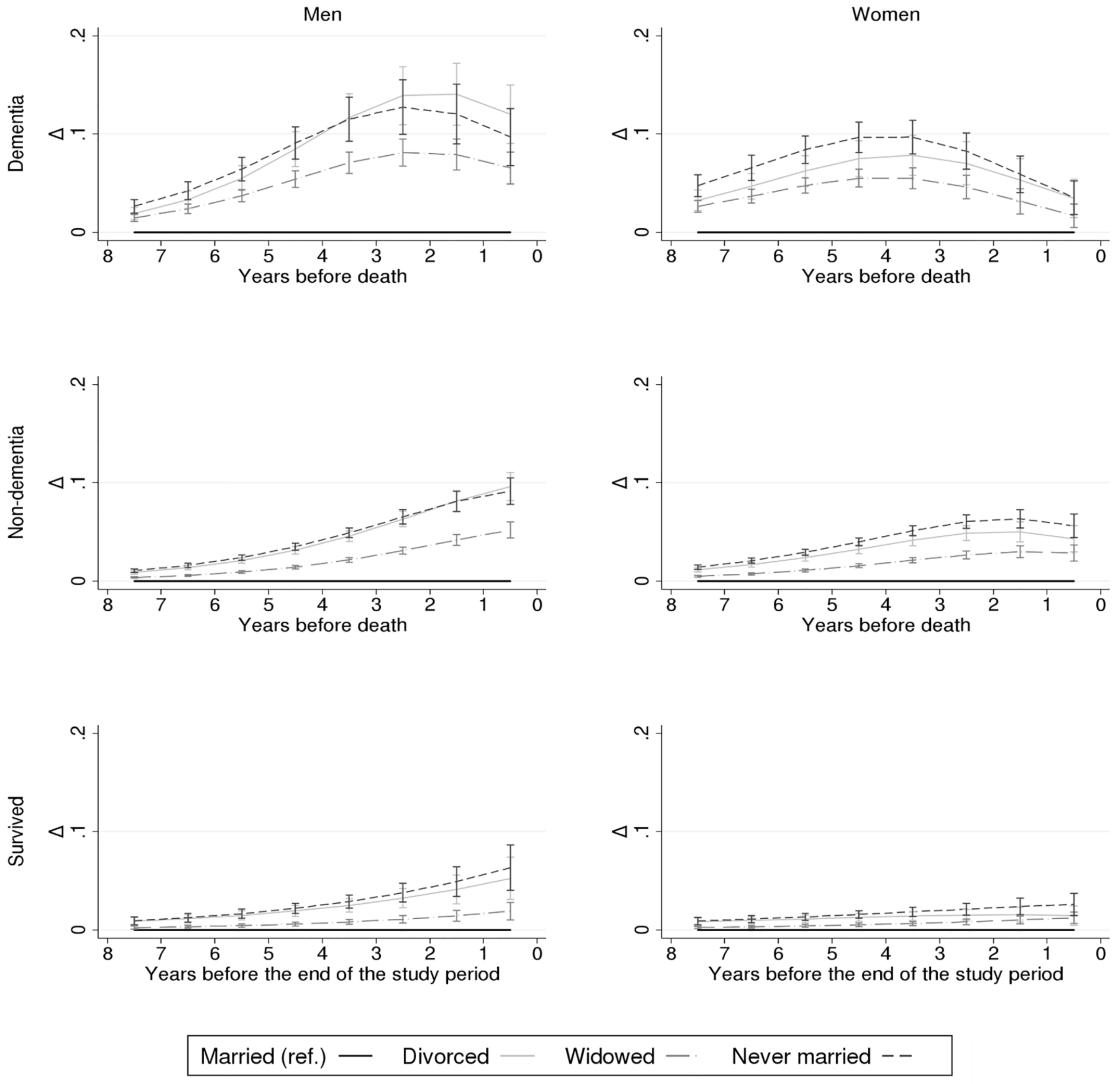
Marital status differences (Δ) in the probability (%) of institutional long-term care (LTC) according to proximity to death (or end of the study period for survivors) and by gender and cause of death; Finnish men and women, 1995–2007. Adjusted for age, household income, calendar year, and region of residence.

Fig 3 shows the differences in the probability of institutional LTC by marital status. In cases of dementia-related death, the difference in the probability between the married and the non-married constantly increased until the fifth year before death among women, and until the second year before death among men. By the last year before death, the differences had decreased to about 6–12 percentage points among men, and disappeared among women. In cases of non-dementia-related death and among the survivors, in contrast, the differences by marital status increased up until death, the pattern being stronger among men than women.

**Fig 4:**
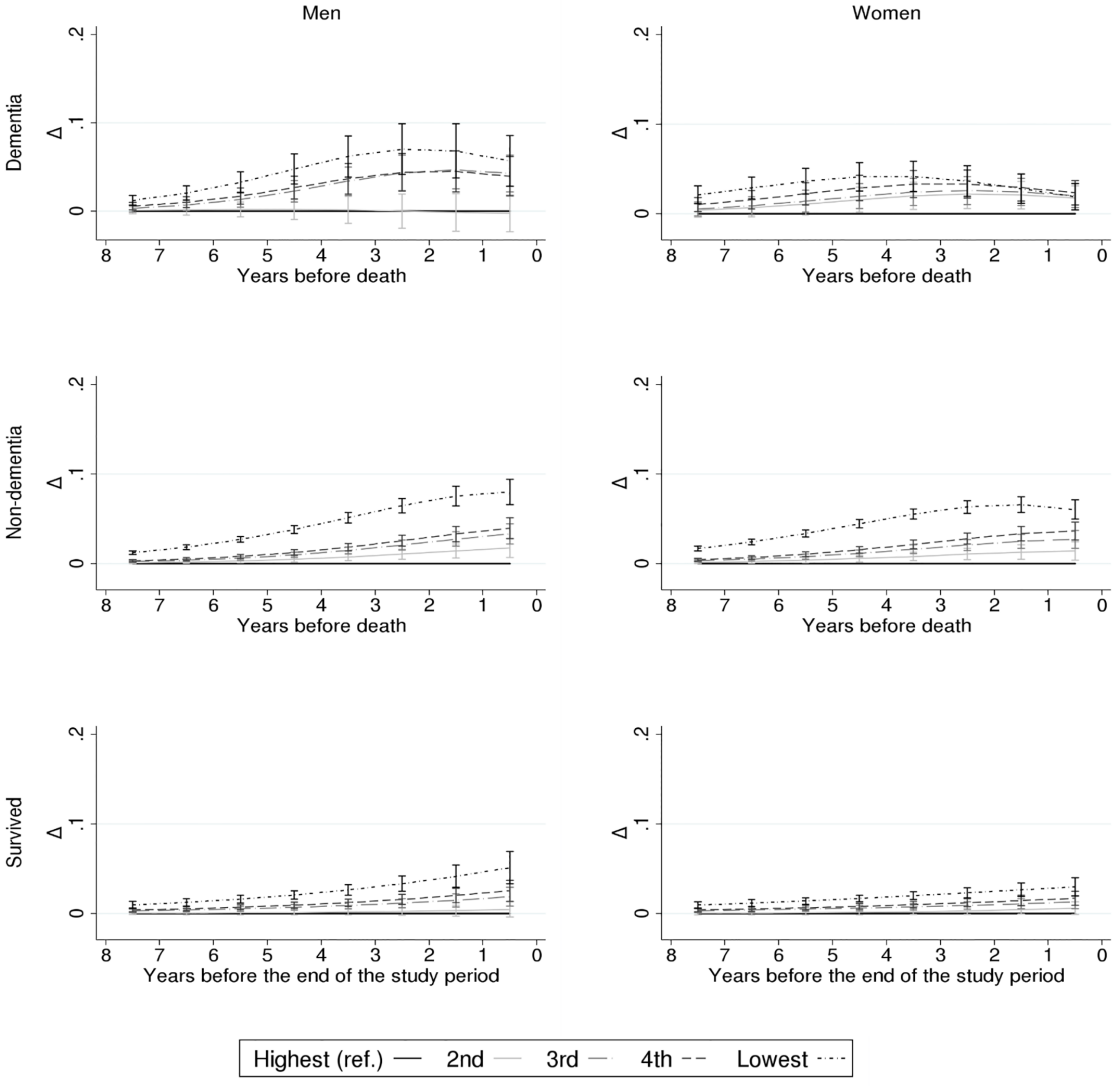
Household income differences (Δ) in the probability (%) of institutional long-term care (LTC) according to proximity to death (or end of the study period for survivors) and by gender and cause of death; Finnish men and women, 1995–2007. Adjusted for age, marital status, calendar year, and region of residence.

Although smaller in magnitude, a similar pattern of diminishing differences before dementia-related death and increasing differences up until non-dementia-related death or end of the study period for survivors emerged for household income (Fig 4). In cases of dementia-related death, the income differences in the probability of institutional LTC disappeared by the last year of life among women, whereas the differences between the highest and lowest income quintiles remained at 5.5 percentage points among men.

## Discussion

### The main results and their interpretation

In a first study of its kind, we investigated the time-varying associations between the use of institutional LTC and socio-demographic and economic factors in the last eight years of life for people who died from dementia. Our results provide support for important socio-demographic and economic differences in institutional LTC among people with dementia; those at older ages and who were unmarried in particular, but also those at lower household incomes, had, on average, a higher risk of institutional LTC. These average associations were similar to people whose death was unrelated to dementia. The socio-demographic and economic differences were not, however, constant throughout the time before death, and the patterns differed between the cause-of-death groups. Among those who died of dementia, the differences peaked at about the third year before death, after which they diminished towards the end of life. In cases of non-dementia-related death, in contrast, the socio-demographic differences continued to increase until the end of life. These findings make two important contributions to the literature. First, the study shows that socio-demographic and economic factors affect the use of institutional LTC among people with dementia but, in the last years of life, people become more equal in this respect. Second, our results indicate that this pattern of increasing and diminishing socio-demographic and economic differences in institutional LTC is unique to dementia.

To our knowledge, no previous studies have investigated time-varying associations between institutional LTC and socio-demographic and economic factors among people with dementia. Our results are, however, in accordance with many of the earlier studies which follow-up individuals with a dementia diagnosis for their first entry into institutional LTC, reporting that older people [11–16], the widowed, and the never married [16,17] have a shorter time to institutionalisation. Other studies [12,16,18] including a systematic review [19], in contrast, report no consistent effects of age, marital status or living arrangements. It is possible that the heterogeneity of previous results relates to the time-varying effects, which have not been identified previously. The age effect may, in some studies, be non-significant due to adjustments for cognitive and functional disabilities, many of which are typical consequences of dementia progression. Based on previous studies, moreover, it has remained unclear whether the higher risk of institutionalisation of older individuals with dementia only reflects shorter survival time of the older [24] or a longer time spent in institutional LTC. With our focus on assessing the use of institutional LTC in the years before death, thus controlling for survival time, we observed a consistent, although diminishing towards the end of life, age effect.

The diminishing effects of socio-demographic and economic factors found in our study are likely to relate to the severe functional and cognitive decline associated with dementia and the considerable burden on informal caregivers. Although socio-demographic and economic differences are substantial 3–4 years before death, they fade in the last years of life, by which time the majority of individuals with dementia are in institutional LTC. Our results show that the differences decline earlier among women than among men, implying that a younger age and having a spouse or a higher income do not necessarily postpone institutional LTC as long for women as perhaps for men. Previous studies on dementia patients [25,26] and the older population in general [27] also report that socio-demographic factors, including marital status and living arrangements, are stronger predictors of entry into institutional LTC among men than among women. Our results also imply that a higher household income may be more beneficial to men than to women in terms of staying in the community, even very close to dementia-related death.

Despite the decreasing influence of socio-demographic factors in the last years of life before death, the results indicate strong associations between institutional LTC and age and marital status among people with dementia. Our analyses show that, when looking at a period several years before death, the differences in institutional LTC by marital status are larger before a dementia-related than non-dementia-related death, reflecting the fact that people with dementia are particularly dependent on informal care. The married are more likely to have a spouse at home providing emotional support and assistance with day-to-day activities, delaying entry into institutional care. A close relationship with the informal caregiver, especially if the spouse is the carer, may also slow the rate of cognitive and functional decline [28]. However, given the limited number of available institutional care places, it is also possible that the local authority, who grants access to institutional LTC, prioritises people who do not have a spouse to help at home. In this case, being married would hinder access to institutional LTC rather than truly support living in the community. To establish the specific mechanisms, further research is warranted.

The income differences in the use of institutional LTC were minor and, for the most part, related to the low income of the non-married. Previously, income differences upon entry into institutional LTC have rarely been investigated among people with dementia, and the few studies that address the question were conducted in the US. These studies reported a higher hazard of institutionalisation for people with a lower income [29] or Medicaid eligibility [13]. Our study provides evidence that differences by income also exist in the Nordic context with publicly provided universal health and social care, although the differences virtually disappear in the last years of life, especially among women.

Nevertheless, small income differences exist, and imply, for example, that people in a lower socio-economic position potentially have a higher number of comorbid conditions [30] and, therefore, a greater need for institutional care. In addition, people with a higher household income may have better access to private-sector care services that help them to cope with dementia and the related functional limitations at home for a longer period. People with a high income may also have an economic incentive to favour other forms of care since the client fees depend on the client’s ability to pay [31]. Municipalities generally charge 85% (80–82% until 2010) of personal disposable income, which is the maximum fee as prescribed by law [32]. The law, however, guarantees that all individuals have at least 100 euros of monthly income available for personal use, thus, people with very low income may pay a smaller share of their disposable income.

### Methodological considerations

Our extensive longitudinal register data allowed us to reliably assess differences between population subgroups in institutional LTC, retrospectively from death over an extended period of time with no selection or attrition bias, problems that are common in clinical samples and most cohort studies. Furthermore, we had access to an annually updated measure of marital status, taking into account the high proportion of individuals becoming widowed during the study period. Despite the high-quality data, however, our study also has some limitations. Since we identified dementia from the Death Register, we could only capture cases where the doctor has recorded dementia as the cause of death. Due to the potential underreporting of dementia as the underlying cause of death [33], we adopted a multiple-cause approach, and also included the cases where dementia was recorded as any of the three contributory causes. The proportion of deaths that we identified as being related to dementia ranged from 5.8% for men and 7.7% for women who died at the age of 70–74, to 22.9% and 31.2%, respectively, for those dying at the age of 90+. These estimates are rather consistent with estimates of dementia prevalence at the time of death reported in Brayne et al. [34] (10% and 44% for those dying at the age of 70–74 and 90–94, respectively), and somewhat higher than the proportion of deaths theoretically attributable to dementia in the UK (3% and 14–19% at the ages of 70–74 and 90–94, respectively) calculated by Knapp et al. [35]. In the Finnish Death Register, the reporting of dementia as a cause of death has improved since the late 1990s, and, moreover, the specificity is extremely high [36]. Thus, we are confident that our measure of dementia does not bias our results.

Another restriction is that we had no information on adult children of the study population. Apart from the spouse, daughters are particularly likely to serve as the informal caregiver [37]. However, we conducted a sensitivity analysis using living arrangements instead of marital status as the explanatory variable, and found that living alone or with others versus living with a marital spouse or cohabiting partner eight years before death increased the probability of institutional LTC in the forthcoming years, but the differences diminished among men and disappeared among women in the last years before a dementia-related death. Thus, our conclusions based on the time-varying measure of marital status appear robust.

### Conclusions

With detailed longitudinal register data our study provides novel evidence on the time-varying associations between institutional LTC and socio-demographic and economic factors prior to dementia-related death. Our results show that being of older age, non-married, and having a lower household income are important predictors of institutional LTC, but decreasingly so in the last years of life among people who die of dementia. Among women in particular, the socio-demographic and economic differences nearly disappear by the end of life. The results indicate that dementia is a burdensome disorder for informal caregivers and often requires intensive professional care once the symptoms have become serious, regardless of socio-demographic and economic resources. Thus, modification of these resources through, for example, delayed widowhood and increasing standard of living are unlikely to offset the substantial increase in the demand for dementia-related institutional LTC related to population ageing.

## Supporting information

S1 Appendix Analytic code. (PDF)
