# Supplementary material for: Time-varying effects of socio-demographic and economic factors on the use of institutional long-term care before dementia-related death: A Finnish register-based study
Source: PLoS One. 2018 Jun 21;13(6):e0199551. doi: 10.1371/journal.pone.0199551 (PMC6013097; doi:10.1371/journal.pone.0199551)
Supplement: S1 Appendix — (PDF) [file pone.0199551.s001.pdf]

```
/** Code by Kaarina Korhonen, 18 May 2018
** Population Research Unit, Faculty of Social Sciences, University of Helsinki
** kaarina.korhonen@helsinki.fi

Analytic code
Time-varying effects of socio-demographic and economic factors on the use of institutional long-term care before dementia-related death: A
Finnish register-based study

Variables used:

ltc (annual dummy):       0) Days in institutional long-term care (LTC) = 0
                          1) Days in LTC >0

cause_of_death:           1) dementia
                          2) non-dementia
                          3) survived

proximity_to_death: years (365-day periods) before death (or end of the study period for survivors), range: 1-8 (inverse coding; 1=the eighth
year before death, 8=the last year before death)

gender:                   1) men
                          2) women

age_group (based on age at death or end of the study period for survivors):                      1) 70-74
                                                                                                  2) 75-79
                                                                                                  3) 80-84
                                                                                                  4) 85-89
                                                                                                  5) 90+
marital_status (time-varying):                  1) married
                                                2) divorced
                                                3) widowed
                                                4) never married

household_income (measured in the 9th-12th year before death or end of the study period for survivors)                  1) Highest quintile
                                                                                                                        2) 2nd
                                                                                                                        3) 3rd
                                                                                                                        4) 4th
                                                                                                                        5) Lowest quintile

id:                       individuals' id

age_at_death:             age at death in years (centered)

age_at_death2:            squared age_at_death

calendar_year:            calendar year at death or end of the study period for survivors, range 2001-2007
```

```
region_of_residence:    region of residence in the 9th year before death or end of the study period for survivors based on county (N=20)

proximity_to_death2:    proximity_to_death squared

proximity_to_death3:    proximity_to_death cubed

*/



/*****************************************************************************************************************
Table 1: Distribution of the study population and unadjusted average annual probability (%) of
institutional long-term care (LTC) use in the eight-year period before death (or end of the study period
for survivors) by age,marital status and household income and by cause of death; Finnish men and women,
1995-2007
*****************************************************************************************************************/


* N by cause of death
            tab cause_of_death if proximity_to_death == 8, m

* % distribution by age group and cause of death
            tab age_group if cause_of_death == 1 & proximity_to_death == 8 [iweight=paino]
            tab age_group if cause_of_death == 2 & proximity_to_death == 8 [iweight=paino]
            tab age_group if cause_of_death == 3 & proximity_to_death == 8 [iweight=paino]


* % distribution of person-years by marital status and cause of death
            tab marital_status if cause_of_death == 1 [iweight=paino]
            tab marital_status if cause_of_death == 2 [iweight=paino]
            tab marital_status if cause_of_death == 3 [iweight=paino]


* % distribution by age group and cause of death
            tab household_income if cause_of_death == 1 & proximity_to_death == 8 [iweight=paino]
            tab household_income if cause_of_death == 2 & proximity_to_death == 8 [iweight=paino]
            tab household_income if cause_of_death == 3 & proximity_to_death == 8 [iweight=paino]


* Unadjusted average annual probability (%) of LTC in the eight-year period before death (or end of the study period)
            bys cause_of_death gender: tab age_group ltc [iweight=paino], row
            bys cause_of_death gender: tab marital_status ltc [iweight=paino], row
            bys cause_of_death gender: tab household_income ltc [iweight=paino], row
```

```
/*****************************************************************************************************
Figure 1: Age-adjusted probability (%) of institutional long-term care (LTC) according to proximity to
death (or the and of the study period for survivors) by gender and cause of death; Finnish men and women,
1995-2007
*****************************************************************************************************/

** Dementia

 * Men
            xtgee ltc c.age_at_death c.age_at_death2 i.proximity_to_death if cause_of_death == 1 & gender == 1 [pweight=npaino], ///
               eform fam(binomial) link(logit) i(id) t(proximity_to_death) corr(ar1)

            margins proximity_to_death, at(age_at_death = (0))  post

            matrix b = e(b)'
            matrix list b
            svmat b, names(est_b)

            matrix v = e(V)
            matrix list v
            matrix se = vecdiag(cholesky(diag(vecdiag(v))))'
            matrix list se

            svmat se, names(se_b)
            clist se_b1 in 1/9

            gen ll_b1 = est_b1 - 1.96*se_b1
            gen ul_b1 = est_b1 + 1.96*se_b1

            clist est_b1-ul_b1 in 1/9

 * Women
            xtgee ltc c.age_at_death c.age_at_death2 i.proximity_to_death if cause_of_death == 1 & gender == 2 [pweight=npaino], ///
               eform fam(binomial) link(logit) i(id) t(proximity_to_death) corr(ar1)

            margins proximity_to_death, at(age_at_death = (0)) post

            matrix b = e(b)'
            matrix list b
            svmat b, names(est_b2)

            matrix v = e(V)
            matrix list v
            matrix se = vecdiag(cholesky(diag(vecdiag(v))))'
            matrix list se

            svmat se, names(se_b2)
            clist se_b2 in 1/9
```

```
            gen ll_b2 = est_b2 - 1.96*se_b2
            gen ul_b2 = est_b2 + 1.96*se_b2

            clist est_b2-ul_b2 in 1/9


** Non-dementia

 * Men
            xtgee ltc c.age_at_death c.age_at_death2 i.proximity_to_death if cause_of_death == 2 & gender == 1 [pweight=npaino], ///
               eform fam(binomial) link(logit) i(id) t(proximity_to_death) corr(ar1)

            margins proximity_to_death, at(age_at_death = (0)) post

            matrix b = e(b)'
            matrix list b
            svmat b, names(est_b3)

            matrix v = e(V)
            matrix list v
            matrix se = vecdiag(cholesky(diag(vecdiag(v))))'
            matrix list se

            svmat se, names(se_b3)
            clist se_b3 in 1/9

            gen ll_b3 = est_b3 - 1.96*se_b3
            gen ul_b3 = est_b3 + 1.96*se_b3

            clist est_b3-ul_b3 in 1/9


 * Women
            xtgee ltc c.age_at_death c.age_at_death2 i.proximity_to_death if cause_of_death == 2 & gender == 2 [pweight=npaino], ///
               eform fam(binomial) link(logit) i(id) t(proximity_to_death) corr(ar1)

            margins proximity_to_death, at(age_at_death = (0)) post

            matrix b = e(b)'
            matrix list b
            svmat b, names(est_b4)

            matrix v = e(V)
            matrix list v
            matrix se = vecdiag(cholesky(diag(vecdiag(v))))'
            matrix list se
```

```
            svmat se, names(se_b4)
            clist se_b4 in 1/9

            gen ll_b4 = est_b4 - 1.96*se_b4
            gen ul_b4 = est_b4 + 1.96*se_b4

            clist est_b4-ul_b4 in 1/9


** Survived

 * Men
            xtgee ltc c.age_at_death c.age_at_death2 i.proximity_to_death if cause_of_death == 3 & gender == 1 [pweight=npaino], ///
               eform fam(binomial) link(logit) i(id) t(proximity_to_death) corr(ar1)

            margins proximity_to_death, at(age_at_death = (0)) post

            matrix b = e(b)'
            matrix list b
            svmat b, names(est_b5)

            matrix v = e(V)
            *matrix v = v[49...,49...]
            matrix list v
            matrix se = vecdiag(cholesky(diag(vecdiag(v))))'
            matrix list se

            svmat se, names(se_b5)
            clist se_b5 in 1/9

            generate ll_b5 = est_b5 - 1.96*se_b5
            generate ul_b5 = est_b5 + 1.96*se_b5

            clist est_b5-ul_b5 in 1/9


 * Women
            xtgee ltc c.age_at_death c.age_at_death2 i.proximity_to_death if cause_of_death == 3 & gender == 2 [pweight=npaino], ///
               eform fam(binomial) link(logit) i(id) t(proximity_to_death) corr(ar1)

            margins proximity_to_death, at(age_at_death = (0))  post

            matrix b = e(b)'
            matrix list b
            svmat b, names(est_b6)

            matrix v = e(V)
            *matrix v = v[49...,49...]
```

```
            matrix list v
            matrix se = vecdiag(cholesky(diag(vecdiag(v))))'
            matrix list se

            svmat se, names(se_b6)
            clist se_b6 in 1/9

            generate ll_b6 = est_b6 - 1.96*se_b6
            generate ul_b6 = est_b6 + 1.96*se_b6

            clist est_b6-ul_b6 in 1/9


** Graph
            gen years_to_death = _n
            replace years_to_death = . if years_to_death > 8

            graph twoway            (line est_b1 years_to_death, lpattern(solid) lcolor(gs10) ) ///
                                    (line est_b2 years_to_death, lpattern(solid) lcolor(gs0) ) ///
                                    (line est_b3 years_to_death, lpattern(longdash) lcolor(gs10)) ///
                                    (line est_b4 years_to_death, lpattern(longdash) lcolor(gs0)) ///
                                    (line est_b5 years_to_death, lpattern(shortdash_dot) lcolor(gs10)) ///
                                    (line est_b6 years_to_death, lpattern(shortdash_dot) lcolor(gs0)) ///
                                      (rcap ll_b1 ul_b1 years_to_death, lpattern(solid) lcolor(gs10) lwidth(thin)) ///
                                      (rcap ll_b2 ul_b2 years_to_death, lpattern(solid) lcolor(gs0) lwidth(thin)) ///
                                      (rcap ll_b3 ul_b3 years_to_death, lpattern(solid) lcolor(gs10) lwidth(thin)) ///
                                      (rcap ll_b4 ul_b4 years_to_death, lpattern(solid) lcolor(gs0) lwidth(thin)) ///
                                      (rcap ll_b5 ul_b5 years_to_death, lpattern(solid) lcolor(gs10) lwidth(thin)) ///
                                      (rcap ll_b6 ul_b6 years_to_death, lpattern(solid) lcolor(gs0) lwidth(thin)), ///
                                    xtitle("Years before death / end of the study period") ///
                                    ytitle("%") ///
                                    xlabel(0.5 "8"  1.5 "7"  2.5 "6" 3.5 "5"  4.5 "4"  5.5 "3"  6.5 "2" 7.5 "1" 8.5 "0", labs(medium) ) ///
                                    ylabel( 0 "0" 0.2 "20" 0.4 "40" 0.6 "60" 0.8 "80"  1 "100" , labs(medium)) ///
                                    graphr(color(white)) ///
                                    legend(order(2 "Women   Dementia" 4 "Non-dementia" 6 "Survived" 1 "Men   Dementia" 3 "Non-dementia" 5
"Survived" ) size(medium) textfirst cols(3) symxsize(6))
```

```
/*****************************************************************************************************************
Table 2: Average annual differences in the probability (%) of institutional long-term care (LTC) and 95%
confidence intervals (CI) in the eight-year period before death (or end of the study period
for survivors) by age, marital status and household income and by cause of death; Finnish men, 1995-2007
*
Table 3: Average annual differences in the probability (%) of institutional long-term care (LTC) and 95%
confidence intervals (CI) in the eight-year period before death (or end of the study period
for survivors) by age, marital status and household income and by cause of death; Finnish women, 1995-2007
*****************************************************************************************************************/



* Age group

 * Dementia

 * Model 1
            xtgee ltc c.calendar_year i.region_of_residence i.age_group i.gender i.age_group#i.gender if cause_of_death == 1 [pweight=npaino],
///
              eform fam(binomial) link(logit) i(id) t(proximity_to_death) corr(ar1)

            margins, dydx(age_group) at(gender = (1 2))  post

            gen n = _n in 1/30

            matrix b = e(b)'
            matrix list b
            matrix b = b[3...,1]
            svmat b, names(est)

            matrix v = e(V)
            matrix list v
            matrix v = v[3...,3...]
            matrix se = vecdiag(cholesky(diag(vecdiag(v))))'
            matrix list se
            svmat se, names(se)
            clist se1 in 1/31

            gen ll = est - 1.96*se1
            gen ul = est + 1.96*se1
            clist est-ul in 1/9

            gen age_groupX = int((mod(_n-1,8))/2)+1 in 1/8
            replace age_groupX = age_groupX + 1
            gen genderX = int((mod(_n-1,2))/1)+1 in 1/8
```

```
 * Model 2
            xtgee ltc c.calendar_year i.region_of_residence i.age_group i.gender i.marital_status i.household_income i.age_group#i.gender if 
cause_of_death == 1 [pweight=npaino], ///
              eform fam(binomial) link(logit) i(id) t(proximity_to_death) corr(ar1)

            margins, dydx(age_group) at(gender = (1 2))  post

            matrix b = e(b)'
            matrix list b
            matrix b = b[3...,1]
            svmat b, names(est2)

            matrix v = e(V)
            matrix list v
            matrix v = v[3...,3...]
            matrix se = vecdiag(cholesky(diag(vecdiag(v))))'
            matrix list se
            svmat se, names(se2)
            clist se2 in 1/31

            generate ll2 = est2 - 1.96*se2
            generate ul2 = est2 + 1.96*se2
            clist est2-ul2 in 1/31


 * Non-dementia

 * Model 1
            xtgee ltc c.calendar_year i.region_of_residence i.age_group i.gender i.age_group#i.gender if cause_of_death == 2 [pweight=npaino], 
///
              eform fam(binomial) link(logit) i(id) t(proximity_to_death) corr(ar1)

            margins, dydx(age_group) at(gender = (1 2))  post

            matrix b = e(b)'
            matrix list b
            matrix b = b[3...,1]
            svmat b, names(est3)

            matrix v = e(V)
            matrix list v
            matrix v = v[3...,3...]
            matrix se = vecdiag(cholesky(diag(vecdiag(v))))'
            matrix list se
            svmat se, names(se3)
            clist se3 in 1/31

            generate ll3 = est3 - 1.96*se3
```

```
            generate ul3 = est3 + 1.96*se3
            clist est3-ul3 in 1/31

 * Model 2
            xtgee ltc c.calendar_year i.region_of_residence i.age_group i.gender i.marital_status i.household_income i.age_group#i.gender if
cause_of_death == 2 [pweight=npaino], ///
              eform fam(binomial) link(logit) i(id) t(proximity_to_death) corr(ar1)

            margins, dydx(age_group) at(gender = (1 2))  post

            matrix b = e(b)'
            matrix list b
            matrix b = b[3...,1]
            svmat b, names(est4)

            matrix v = e(V)
            matrix list v
            matrix v = v[3...,3...]
            matrix se = vecdiag(cholesky(diag(vecdiag(v))))'
            matrix list se
            svmat se, names(se4)
            clist se4 in 1/31

            generate ll4 = est4 - 1.96*se4
            generate ul4 = est4 + 1.96*se4
            clist est4-ul4 in 1/31


 * Survived

 * Model 1
            xtgee ltc c.calendar_year i.region_of_residence i.age_group i.gender i.age_group#i.gender if cause_of_death == 3 [pweight=npaino],
///
              eform fam(binomial) link(logit) i(id) t(proximity_to_death) corr(ar1)

            margins, dydx(age_group) at(gender = (1 2))  post

            matrix b = e(b)'
            matrix list b
            matrix b = b[3...,1]
            svmat b, names(est5)

            matrix v = e(V)
            matrix list v
            matrix v = v[3...,3...]
            matrix se = vecdiag(cholesky(diag(vecdiag(v))))'
            matrix list se
            svmat se, names(se5)
```

```
            clist se5 in 1/31

            generate ll5 = est5 - 1.96*se5
            generate ul5 = est5 + 1.96*se5
            clist est5-ul5 in 1/31

 * Model 2
            xtgee ltc c.calendar_year i.region_of_residence i.age_group i.gender i.marital_status i.household_income i.age_group#i.gender if
cause_of_death == 3 [pweight=npaino], ///
              eform fam(binomial) link(logit) i(id) t(proximity_to_death) corr(ar1)

            margins, dydx(age_group) at(gender = (1 2))  post

            matrix b = e(b)'
            matrix list b
            matrix b = b[3...,1]
            svmat b, names(est6)

            matrix v = e(V)
            matrix list v
            matrix v = v[3...,3...]
            matrix se = vecdiag(cholesky(diag(vecdiag(v))))'
            matrix list se
            svmat se, names(se6)
            clist se6 in 1/31

            generate ll6 = est6 - 1.96*se6
            generate ul6 = est6 + 1.96*se6
            clist est6-ul6 in 1/31

* Export to excel
            sort genderX age_groupX
            export excel n genderX age_groupX est1 ll ul est2 ll2 ul2 est3 ll3 ul3 est4 ll4 ul4 est5 ll5 ul5 est6 ll6 ul6 if n < 9 using "...
/table2_3_age_group.xlsx", replace
            sort n


* Marital status

 * Dementia

 * Model 1
            xtgee ltc c.calendar_year i.region_of_residence c.age_at_death c.age_at_death2 i.gender i.marital_status i.marital_status#i.gender
if cause_of_death == 1 [pweight=npaino], ///
              eform fam(binomial) link(logit) i(id) t(proximity_to_death) corr(ar1)

            margins, dydx(marital_status) at(age_at_death = 0 gender = (1 2))  post
```

```
            matrix b = e(b)'
            matrix list b
            matrix b = b[3...,1]
            svmat b, names(est7)

            matrix v = e(V)
            matrix list v
            matrix v = v[3...,3...]
            matrix se = vecdiag(cholesky(diag(vecdiag(v))))'
            matrix list se
            svmat se, names(se7)
            clist se7 in 1/25

            generate ll7 = est7 - 1.96*se7
            generate ul7 = est7 + 1.96*se7
            clist est7-ul7 in 1/25

            gen marital_statusX = int((mod(_n-1,6))/2)+1 in 1/6
            replace marital_statusX = marital_statusX + 1

 * Model 2
            xtgee ltc c.calendar_year i.region_of_residence c.age_at_death c.age_at_death2 i.gender i.marital_status i.household_income
i.marital_status#i.gender if cause_of_death == 1 [pweight=npaino], ///
              eform fam(binomial) link(logit) i(id) t(proximity_to_death) corr(ar1)

            margins, dydx(marital_status) at(age_at_death = 0 gender = (1 2))  post

            matrix b = e(b)'
            matrix list b
            matrix b = b[3...,1]
            svmat b, names(est8)

            matrix v = e(V)
            matrix list v
            matrix v = v[3...,3...]
            matrix se = vecdiag(cholesky(diag(vecdiag(v))))'
            matrix list se
            svmat se, names(se8)
            clist se8 in 1/25

            generate ll8 = est8 - 1.96*se8
            generate ul8 = est8 + 1.96*se8
            clist est8-ul8 in 1/25



 * Non-dementia
```

```
 * Model 1
            xtgee ltc c.calendar_year i.region_of_residence c.age_at_death c.age_at_death2 i.gender i.marital_status i.marital_status#i.gender
if cause_of_death == 2 [pweight=npaino], ///
              eform fam(binomial) link(logit) i(id) t(proximity_to_death) corr(ar1)

            margins, dydx(marital_status) at(age_at_death = 0 gender = (1 2))  post

            matrix b = e(b)'
            matrix list b
            matrix b = b[3...,1]
            svmat b, names(est9)

            matrix v = e(V)
            matrix list v
            matrix v = v[3...,3...]
            matrix se = vecdiag(cholesky(diag(vecdiag(v))))'
            matrix list se
            svmat se, names(se9)
            clist se9 in 1/25

            generate ll9 = est9 - 1.96*se9
            generate ul9 = est9 + 1.96*se9
            clist est9-ul9 in 1/25

 * Model 2
            xtgee ltc c.calendar_year i.region_of_residence c.age_at_death c.age_at_death2 i.gender i.marital_status i.household_income
i.marital_status#i.gender if cause_of_death == 2 [pweight=npaino], ///
              eform fam(binomial) link(logit) i(id) t(proximity_to_death) corr(ar1)

            margins, dydx(marital_status) at(age_at_death = 0 gender = (1 2))  post

            matrix b = e(b)'
            matrix list b
            matrix b = b[3...,1]
            svmat b, names(est10)

            matrix v = e(V)
            matrix list v
            matrix v = v[3...,3...]
            matrix se = vecdiag(cholesky(diag(vecdiag(v))))'
            matrix list se
            svmat se, names(se10)
            clist se10 in 1/25

            generate ll10 = est10 - 1.96*se10
            generate ul10 = est10 + 1.96*se10
            clist est10-ul10 in 1/25
```

```
 * Survived

 * Model 1
            xtgee ltc c.calendar_year i.region_of_residence c.age_at_death c.age_at_death2 i.gender i.marital_status i.marital_status#i.gender
if cause_of_death == 3 [pweight=npaino], ///
              eform fam(binomial) link(logit) i(id) t(proximity_to_death) corr(ar1)

            margins, dydx(marital_status) at(age_at_death = 0 gender = (1 2))  post

            matrix b = e(b)'
            matrix list b
            matrix b = b[3...,1]
            svmat b, names(est11)

            matrix v = e(V)
            matrix list v
            matrix v = v[3...,3...]
            matrix se = vecdiag(cholesky(diag(vecdiag(v))))'
            matrix list se
            svmat se, names(se11)
            clist se11 in 1/25

            generate ll11 = est11 - 1.96*se11
            generate ul11 = est11 + 1.96*se11
            clist est11-ul11 in 1/25

 * Model 2
            xtgee ltc c.calendar_year i.region_of_residence c.age_at_death c.age_at_death2 i.gender i.marital_status i.household_income
i.marital_status#i.gender if cause_of_death == 3 [pweight=npaino], ///
              eform fam(binomial) link(logit) i(id) t(proximity_to_death) corr(ar1)

            margins, dydx(marital_status) at(age_at_death = 0 gender = (1 2))  post

            matrix b = e(b)'
            matrix list b
            matrix b = b[3...,1]
            svmat b, names(est12)

            matrix v = e(V)
            matrix list v
            matrix v = v[3...,3...]
            matrix se = vecdiag(cholesky(diag(vecdiag(v))))'
            matrix list se
            svmat se, names(se12)
            clist se12 in 1/25
```

```
            generate ll12 = est12 - 1.96*se12
            generate ul12 = est12 + 1.96*se12
            clist est12-ul12 in 1/25

* Export to excel
            sort genderX marital_statusX
            export excel n genderX marital_statusX est7 ll7 ul7 est8 ll8 ul8 est9 ll9 ul9 est10 ll10 ul10 est11 ll11 ul11 est12 ll12 ul12 if n 
< 8 using ".../table2_3_marital_status.xlsx", replace
            sort n


* Household income

 * Dementia

 * Model 1
            xtgee ltc c.calendar_year i.region_of_residence c.age_at_death c.age_at_death2 i.gender i.household_income 
i.household_income#i.gender if cause_of_death == 1 [pweight=npaino], ///
              eform fam(binomial) link(logit) i(id) t(proximity_to_death) corr(ar1)

            margins, dydx(household_income) at(age_at_death = 0 gender = (1 2)) post

            matrix b = e(b)'
            matrix list b
            matrix b = b[3...,1]
            svmat b, names(est13)

            matrix v = e(V)
            matrix list v
            matrix v = v[3...,3...]
            matrix se = vecdiag(cholesky(diag(vecdiag(v))))'
            matrix list se
            svmat se, names(se13)
            clist se13 in 1/31

            generate ll13 = est13 - 1.96*se13
            generate ul13 = est13 + 1.96*se13
            clist est13-ul13 in 1/31

            gen household_incomeX = int((mod(_n-1,8))/2)+1 in 1/8
            replace household_incomeX = household_incomeX + 1

 * Model 2
            xtgee ltc c.calendar_year i.region_of_residence c.age_at_death c.age_at_death2 i.gender i.marital_status i.household_income 
i.household_income#i.gender if cause_of_death == 1 [pweight=npaino], ///
              eform fam(binomial) link(logit) i(id) t(proximity_to_death) corr(ar1)

            margins, dydx(household_income) at(age_at_death = 0 gender = (1 2)) post
```

```
            matrix b = e(b)'
            matrix list b
            matrix b = b[3...,1]
            svmat b, names(est14)

            matrix v = e(V)
            matrix list v
            matrix v = v[3...,3...]
            matrix se = vecdiag(cholesky(diag(vecdiag(v))))'
            matrix list se
            svmat se, names(se14)
            clist se14 in 1/31

            generate ll14 = est14 - 1.96*se14
            generate ul14 = est14 + 1.96*se14
            clist est14-ul14 in 1/31



 * Non-dementia

 * Model 1
            xtgee ltc c.calendar_year i.region_of_residence c.age_at_death c.age_at_death2 i.gender i.household_income
i.household_income#i.gender if cause_of_death == 2 [pweight=npaino], ///
              eform fam(binomial) link(logit) i(id) t(proximity_to_death) corr(ar1)

            margins, dydx(household_income) at(age_at_death = 0 gender = (1 2)) post

            matrix b = e(b)'
            matrix list b
            matrix b = b[3...,1]
            svmat b, names(est15)

            matrix v = e(V)
            matrix list v
            matrix v = v[3...,3...]
            matrix se = vecdiag(cholesky(diag(vecdiag(v))))'
            matrix list se
            svmat se, names(se15)
            clist se15 in 1/31

            generate ll15 = est15 - 1.96*se15
            generate ul15 = est15 + 1.96*se15
            clist est15-ul15 in 1/31

 * Model 2
```

```
            xtgee ltc c.calendar_year i.region_of_residence c.age_at_death c.age_at_death2 i.gender i.marital_status i.household_income
i.household_income#i.gender if cause_of_death == 2 [pweight=npaino], ///
              eform fam(binomial) link(logit) i(id) t(proximity_to_death) corr(ar1)

            margins, dydx(household_income) at(age_at_death = 0 gender = (1 2)) post

            matrix b = e(b)'
            matrix list b
            matrix b = b[3...,1]
            svmat b, names(est16)

            matrix v = e(V)
            matrix list v
            matrix v = v[3...,3...]
            matrix se = vecdiag(cholesky(diag(vecdiag(v))))'
            matrix list se
            svmat se, names(se16)
            clist se16 in 1/31

            generate ll16 = est16 - 1.96*se16
            generate ul16 = est16 + 1.96*se16
            clist est16-ul16 in 1/31


 * Survived

 * Model 1
            xtgee ltc c.calendar_year i.region_of_residence c.age_at_death c.age_at_death2 i.gender i.household_income
i.household_income#i.gender if cause_of_death == 3 [pweight=npaino], ///
              eform fam(binomial) link(logit) i(id) t(proximity_to_death) corr(ar1)

            margins, dydx(household_income) at(age_at_death = 0 gender = (1 2)) post

            matrix b = e(b)'
            matrix list b
            matrix b = b[3...,1]
            svmat b, names(est17)

            matrix v = e(V)
            matrix list v
            matrix v = v[3...,3...]
            matrix se = vecdiag(cholesky(diag(vecdiag(v))))'
            matrix list se
            svmat se, names(se17)
            clist se17 in 1/31

            generate ll17 = est17 - 1.96*se17
            generate ul17 = est17 + 1.96*se17
```

```
            clist est17-ul17 in 1/31

 * Model 2
            xtgee ltc c.calendar_year i.region_of_residence c.age_at_death c.age_at_death2 i.gender i.marital_status i.household_income 
i.household_income#i.gender if cause_of_death == 3 [pweight=npaino], ///
              eform fam(binomial) link(logit) i(id) t(proximity_to_death) corr(ar1)

            margins, dydx(household_income) at(age_at_death = 0 gender = (1 2)) post

            matrix b = e(b)'
            matrix list b
            matrix b = b[3...,1]
            svmat b, names(est18)

            matrix v = e(V)
            matrix list v
            matrix v = v[3...,3...]
            matrix se = vecdiag(cholesky(diag(vecdiag(v))))'
            matrix list se
            svmat se, names(se18)
            clist se18 in 1/31

            generate ll18 = est18 - 1.96*se18
            generate ul18 = est18 + 1.96*se18
            clist est18-ul18 in 1/31

* Export to excel
            sort genderX household_incomeX
            export excel n genderX household_incomeX est13 ll13 ul13 est14 ll14 ul14 est15 ll15 ul15 est16 ll16 ul16 est17 ll17 ul17 est18 
ll18 ul18 if n < 9 using ".../table2_3_household_income.xlsx", replace
            sort n



/*****************************************************************************************************************
Figure 2: Age differences in the probability (%) of institutional long-term care (LTC) according to proximity
to death (or the and of the study period for survivors) and by gender and cause of death; Finnish men and women, 
1995-2007
*****************************************************************************************************************/



* Fit the models and save results
            xtgee ltc c.calendar_year i.region_of_residence i.age_group i.gender i.marital_status i.household_income c.proximity_to_death 
c.proximity_to_death2 c.proximity_to_death3 c.proximity_to_death#i.age_group#i.gender if cause_of_death == 1 [pweight=npaino], ///
              eform fam(binomial) link(logit) i(id) t(proximity_to_death) corr(ar1)
            est store agegroup1
```

```
            xtgee ltc c.calendar_year i.region_of_residence i.age_group i.gender i.marital_status i.household_income c.proximity_to_death
c.proximity_to_death2 c.proximity_to_death3 c.proximity_to_death#i.age_group#i.gender if cause_of_death == 2 [pweight=npaino], ///
              eform fam(binomial) link(logit) i(id) t(proximity_to_death) corr(ar1)
            est store agegroup2

            xtgee ltc c.calendar_year i.region_of_residence i.age_group i.gender i.marital_status i.household_income c.proximity_to_death
c.proximity_to_death2 c.proximity_to_death3 c.proximity_to_death#i.age_group#i.gender if cause_of_death == 3 [pweight=npaino], ///
              eform fam(binomial) link(logit) i(id) t(proximity_to_death) corr(ar1)
            est store agegroup3

* Estimate marginal effects

            est restore agegroup1
            margins, dydx(age_group) at(gender = (1 2) proximity_to_death = (1 2 3 4 5 6 7 8)) post

            gen n = _n in 1/64
            gen yline = 0 in 1/64

            matrix b = e(b)'
            matrix list b
            matrix b = b[17...,1]
            svmat b, names(est)

            matrix v = e(V)
            matrix list v
            matrix v = v[17...,17...]
            matrix se = vecdiag(cholesky(diag(vecdiag(v))))'
            matrix list se
            svmat se, names(se)
            clist se1 in 1/64

            gen ll = est - 1.96*se1
            gen ul = est + 1.96*se1
            clist est-ul in 1/65

            gen age_groupF = int((mod(_n-1,64))/16)+1 in 1/64
            replace age_groupF = age_groupF + 1
            gen genderF = int((mod(_n-1,16))/8)+1 in 1/64
            gen proximity_to_deathF = int((mod(_n-1,8))/1)+1 in 1/64


            est restore agegroup2
            margins, dydx(age_group) at(gender = (1 2) proximity_to_death = (1 2 3 4 5 6 7 8)) post

            matrix b = e(b)'
            matrix list b
            matrix b = b[17...,1]
```

```
svmat b, names(est2)

matrix v = e(V)
matrix list v
matrix v = v[17...,17...]
matrix se = vecdiag(cholesky(diag(vecdiag(v))))'
matrix list se
svmat se, names(se2)
clist se2 in 1/64

generate ll2 = est2 - 1.96*se2
generate ul2 = est2 + 1.96*se2
clist est2-ul2 in 1/65


est restore agegroup3
margins, dydx(age_group) at(gender = (1 2) proximity_to_death = (1 2 3 4 5 6 7 8)) post

matrix b = e(b)'
matrix list b
matrix b = b[17...,1]
svmat b, names(est3)

matrix v = e(V)
matrix list v
matrix v = v[17...,17...]
matrix se = vecdiag(cholesky(diag(vecdiag(v))))'
matrix list se
svmat se, names(se3)
clist se3 in 1/64

generate ll3 = est3 - 1.96*se3
generate ul3 = est3 + 1.96*se3
clist est3-ul3 in 1/65


graph twoway ///
(line yline proximity_to_deathF, lpattern(solid) lcolor(gs0)) ///
(line est1 proximity_to_deathF if age_groupF == 2 & genderF == 1, lpattern(solid) lcolor(gs12) ) ///
(line est1 proximity_to_deathF if age_groupF == 3 & genderF == 1, lpattern(longdash_dot) lcolor(gs8)) ///
(line est1 proximity_to_deathF if age_groupF == 4 & genderF == 1, lpattern(dash) lcolor(gs4)) ///
(line est1 proximity_to_deathF if age_groupF == 5 & genderF == 1, lpattern(shortdash_dot) lcolor(gs0)) ///

  (rcap ll ul proximity_to_deathF if age_groupF == 2 & genderF == 1, lpattern(solid) lcolor(gs12) lwidth(thin))          ///
  (rcap ll ul proximity_to_deathF if age_groupF == 3 & genderF == 1, lpattern(solid) lcolor(gs8) lwidth(thin)) ///
  (rcap ll ul proximity_to_deathF if age_groupF == 4 & genderF == 1, lpattern(solid) lcolor(gs4) lwidth(thin)) ///
  (rcap ll ul proximity_to_deathF if age_groupF == 5 & genderF == 1, lpattern(solid) lcolor(gs0) lwidth(thin)), ///
            xtitle("Years before death", size(large)) ///
```

```
                    xlabel(0.5 "8"  1.5 "7"  2.5 "6" 3.5 "5"  4.5 "4"  5.5 "3"  6.5 "2" 7.5 "1" 8.5 "0", labs(large) ) ///
                    ytitle("Dementia" " " "â^†", size(large)) ///
                    ylabel( 0(0.2)0.4, labs(large)) ///
                    yscale(range(-0.02/0.4)) ///
                    graphr(color(white)) ///
                    legend(order(1 "70-74 (ref.)" 2 "75-79" 3 "80-84" 4 "85-89" 5 "90+") size(large) textfirst cols(5) symxsize(6)
region(col(white))) ///
                    subtitle("Men", size(large)) ///
                    aspect(0.5) ///
                    name(fig2_1, replace)

            graph twoway ///
            (line yline proximity_to_deathF, lpattern(solid) lcolor(gs0)) ///
            (line est1 proximity_to_deathF if age_groupF == 2 & genderF == 2, lpattern(solid) lcolor(gs12) ) ///
            (line est1 proximity_to_deathF if age_groupF == 3 & genderF == 2, lpattern(longdash_dot) lcolor(gs8)) ///
            (line est1 proximity_to_deathF if age_groupF == 4 & genderF == 2, lpattern(dash) lcolor(gs4)) ///
            (line est1 proximity_to_deathF if age_groupF == 5 & genderF == 2, lpattern(shortdash_dot) lcolor(gs0)) ///

              (rcap ll ul proximity_to_deathF if age_groupF == 2 & genderF == 2, lpattern(solid) lcolor(gs12) lwidth(thin))          ///
              (rcap ll ul proximity_to_deathF if age_groupF == 3 & genderF == 2, lpattern(solid) lcolor(gs8) lwidth(thin)) ///
              (rcap ll ul proximity_to_deathF if age_groupF == 4 & genderF == 2, lpattern(solid) lcolor(gs4) lwidth(thin)) ///
              (rcap ll ul proximity_to_deathF if age_groupF == 5 & genderF == 2, lpattern(solid) lcolor(gs0) lwidth(thin)), ///
                    xtitle("Years before death", size(large)) ///
                    xlabel(0.5 "8"  1.5 "7"  2.5 "6" 3.5 "5"  4.5 "4"  5.5 "3"  6.5 "2" 7.5 "1" 8.5 "0", labs(large) ) ///
                    ytitle(" " " " "â^†", size(large)) ///
                    ylabel( 0(0.2)0.4, labs(large))     ///
                    yscale(range(-0.02/0.4)) ///
                    graphr(color(white)) ///
                    legend(order(1 "70-74 (ref.)" 2 "75-79" 3 "80-84" 4 "85-89" 5 "90+") size(large) textfirst cols(5) symxsize(6)
region(col(white))) ///
                    subtitle("Women", size(large)) ///
                    aspect(0.5) ///
                    name(fig2_2, replace)

            graph twoway ///
            (line yline proximity_to_deathF, lpattern(solid) lcolor(gs0)) ///
            (line est2 proximity_to_deathF if age_groupF == 2 & genderF == 1, lpattern(solid) lcolor(gs12) ) ///
            (line est2 proximity_to_deathF if age_groupF == 3 & genderF == 1, lpattern(longdash_dot) lcolor(gs8)) ///
            (line est2 proximity_to_deathF if age_groupF == 4 & genderF == 1, lpattern(dash) lcolor(gs4)) ///
            (line est2 proximity_to_deathF if age_groupF == 5 & genderF == 1, lpattern(shortdash_dot) lcolor(gs0)) ///

              (rcap ll2 ul2 proximity_to_deathF if age_groupF == 2 & genderF == 1, lpattern(solid) lcolor(gs12) lwidth(thin)) ///
              (rcap ll2 ul2 proximity_to_deathF if age_groupF == 3 & genderF == 1, lpattern(solid) lcolor(gs8) lwidth(thin)) ///
              (rcap ll2 ul2 proximity_to_deathF if age_groupF == 4 & genderF == 1, lpattern(solid) lcolor(gs4) lwidth(thin)) ///
              (rcap ll2 ul2 proximity_to_deathF if age_groupF == 5 & genderF == 1, lpattern(solid) lcolor(gs0) lwidth(thin)), ///
                    xtitle("Years before death", size(large)) ///
                    xlabel(0.5 "8"  1.5 "7"  2.5 "6" 3.5 "5"  4.5 "4"  5.5 "3"  6.5 "2" 7.5 "1" 8.5 "0", labs(large) ) ///
                    ytitle("Non-dementia" " " "â^†", size(large)) ///
```

```
                    ylabel( 0(0.2)0.4, labs(large))     ///
                    yscale(range(-0.02/0.4)) ///
                    graphr(color(white)) ///
                    legend(order(1 "70-74 (ref.)" 2 "75-79" 3 "80-84" 4 "85-89" 5 "90+") size(large) textfirst cols(5) symxsize(6)
region(col(white))) ///
                    subtitle(" ", size(large)) ///
                    aspect(0.5) ///
                    name(fig2_3, replace)

            graph twoway ///
            (line yline proximity_to_deathF, lpattern(solid) lcolor(gs0)) ///
            (line est2 proximity_to_deathF if age_groupF == 2 & genderF == 2, lpattern(solid) lcolor(gs12) ) ///
            (line est2 proximity_to_deathF if age_groupF == 3 & genderF == 2, lpattern(longdash_dot) lcolor(gs8)) ///
            (line est2 proximity_to_deathF if age_groupF == 4 & genderF == 2, lpattern(dash) lcolor(gs4)) ///
            (line est2 proximity_to_deathF if age_groupF == 5 & genderF == 2, lpattern(shortdash_dot) lcolor(gs0)) ///

              (rcap ll2 ul2 proximity_to_deathF if age_groupF == 2 & genderF == 2, lpattern(solid) lcolor(gs12) lwidth(thin)) ///
              (rcap ll2 ul2 proximity_to_deathF if age_groupF == 3 & genderF == 2, lpattern(solid) lcolor(gs8) lwidth(thin)) ///
              (rcap ll2 ul2 proximity_to_deathF if age_groupF == 4 & genderF == 2, lpattern(solid) lcolor(gs4) lwidth(thin)) ///
              (rcap ll2 ul2 proximity_to_deathF if age_groupF == 5 & genderF == 2, lpattern(solid) lcolor(gs0) lwidth(thin)), ///
                    xtitle("Years before death", size(large)) ///
                    xlabel(0.5 "8"  1.5 "7"  2.5 "6" 3.5 "5"  4.5 "4"  5.5 "3"  6.5 "2" 7.5 "1" 8.5 "0", labs(large) ) ///
                    ytitle(" " " " "âˆ†", size(large)) ///
                    ylabel( 0(0.2)0.4, labs(large))     ///
                    yscale(range(-0.02/0.4)) ///
                    graphr(color(white)) ///
                    legend(order(1 "70-74 (ref.)" 2 "75-79" 3 "80-84" 4 "85-89" 5 "90+") size(large) textfirst cols(5) symxsize(6)
region(col(white))) ///
                    subtitle(" ", size(large)) ///
                    aspect(0.5) ///
                    name(fig2_4, replace)

            graph twoway ///
            (line yline proximity_to_deathF, lpattern(solid) lcolor(gs0)) ///
            (line est3 proximity_to_deathF if age_groupF == 2 & genderF == 1, lpattern(solid) lcolor(gs12) ) ///
            (line est3 proximity_to_deathF if age_groupF == 3 & genderF == 1, lpattern(longdash_dot) lcolor(gs8)) ///
            (line est3 proximity_to_deathF if age_groupF == 4 & genderF == 1, lpattern(dash) lcolor(gs4)) ///
            (line est3 proximity_to_deathF if age_groupF == 5 & genderF == 1, lpattern(shortdash_dot) lcolor(gs0)) ///

              (rcap ll3 ul3 proximity_to_deathF if age_groupF == 2 & genderF == 1, lpattern(solid) lcolor(gs12) lwidth(thin)) ///
              (rcap ll3 ul3 proximity_to_deathF if age_groupF == 3 & genderF == 1, lpattern(solid) lcolor(gs8) lwidth(thin)) ///
              (rcap ll3 ul3 proximity_to_deathF if age_groupF == 4 & genderF == 1, lpattern(solid) lcolor(gs4) lwidth(thin)) ///
              (rcap ll3 ul3 proximity_to_deathF if age_groupF == 5 & genderF == 1, lpattern(solid) lcolor(gs0) lwidth(thin)), ///
                    xtitle("Years before the end of the study period", size(large))         ///
                    xlabel(0.5 "8"  1.5 "7"  2.5 "6" 3.5 "5"  4.5 "4"  5.5 "3"  6.5 "2" 7.5 "1" 8.5 "0", labs(large) ) ///
                    ytitle("Survived" " " "âˆ†", size(large)) ///
                    ylabel( 0(0.2)0.4, labs(large)) ///
                    yscale(range(-0.02/0.4)) ///
```

```
                        graphr(color(white)) ///
                        legend(order(1 "70-74 (ref.)" 2 "75-79" 3 "80-84" 4 "85-89" 5 "90+") size(large) textfirst cols(5) symxsize(6)
region(col(white))) ///
                        subtitle(" ", size(large)) ///
                        aspect(0.5) ///
                        name(fig2_5, replace)

            graph twoway ///
            (line yline proximity_to_deathF, lpattern(solid) lcolor(gs0)) ///
            (line est3 proximity_to_deathF if age_groupF == 2 & genderF == 2, lpattern(solid) lcolor(gs12) ) ///
            (line est3 proximity_to_deathF if age_groupF == 3 & genderF == 2, lpattern(longdash_dot) lcolor(gs8)) ///
            (line est3 proximity_to_deathF if age_groupF == 4 & genderF == 2, lpattern(dash) lcolor(gs4)) ///
            (line est3 proximity_to_deathF if age_groupF == 5 & genderF == 2, lpattern(shortdash_dot) lcolor(gs0)) ///

              (rcap ll3 ul3 proximity_to_deathF if age_groupF == 2 & genderF == 2, lpattern(solid) lcolor(gs12) lwidth(thin)) ///
              (rcap ll3 ul3 proximity_to_deathF if age_groupF == 3 & genderF == 2, lpattern(solid) lcolor(gs8) lwidth(thin)) ///
              (rcap ll3 ul3 proximity_to_deathF if age_groupF == 4 & genderF == 2, lpattern(solid) lcolor(gs4) lwidth(thin)) ///
              (rcap ll3 ul3 proximity_to_deathF if age_groupF == 5 & genderF == 2, lpattern(solid) lcolor(gs0) lwidth(thin)), ///
                        xtitle("Years before the end of the study period", size(large))         ///
                        xlabel(0.5 "8"  1.5 "7"  2.5 "6" 3.5 "5"  4.5 "4"  5.5 "3"  6.5 "2" 7.5 "1" 8.5 "0", labs(large) ) ///
                        ytitle(" " " " "â^†", size(large)) ///
                        ylabel( 0(0.2)0.4, labs(large))     ///
                        yscale(range(-0.02/0.4)) ///
                        graphr(color(white)) ///
                        legend(order(1 "70-74 (ref.)" 2 "75-79" 3 "80-84" 4 "85-89" 5 "90+") size(large) textfirst cols(5) symxsize(6)
region(col(white))) ///
                        subtitle(" ", size(large)) ///
                        aspect(0.5) ///
                        name(fig2_6, replace)
```

```
/*****************************************************************************************************************
Figure 3: Marital status differences in the probability (%) of institutional long-term care (LTC) according to proximity
to death (or the and of the study period for survivors) and by gender and cause of death; Finnish men and women,
1995-2007
*****************************************************************************************************************/

* Fit the models and save results

            xtgee ltc c.calendar_year i.region_of_residence c.age_at_death c.age_at_death2 i.gender i.marital_status i.household_income
c.proximity_to_death c.proximity_to_death2 c.proximity_to_death3 c.proximity_to_death#i.marital_status#i.gender if cause_of_death == 1
[pweight=npaino], ///
              eform fam(binomial) link(logit) i(id) t(proximity_to_death) corr(ar1)
            est store maritalstatus1

            xtgee ltc c.calendar_year i.region_of_residence c.age_at_death c.age_at_death2 i.gender i.marital_status i.household_income
c.proximity_to_death c.proximity_to_death2 c.proximity_to_death3 c.proximity_to_death#i.marital_status#i.gender if cause_of_death == 2
[pweight=npaino], ///
              eform fam(binomial) link(logit) i(id) t(proximity_to_death) corr(ar1)
            est store maritalstatus2

            xtgee ltc c.calendar_year i.region_of_residence c.age_at_death c.age_at_death2 i.gender i.marital_status i.household_income
c.proximity_to_death c.proximity_to_death2 c.proximity_to_death3 c.proximity_to_death#i.marital_status#i.gender if cause_of_death == 3
[pweight=npaino], ///
              eform fam(binomial) link(logit) i(id) t(proximity_to_death) corr(ar1)
            est store maritalstatus3


* Estimate marginal effects

            est restore maritalstatus1
            margins, dydx(marital_status) at(age_at_death = 0 gender = (1 2) proximity_to_death = (1 2 3 4 5 6 7 8)) post

            matrix b = e(b)'
            matrix list b
            matrix b = b[17...,1]
            svmat b, names(est4)

            matrix v = e(V)
            matrix list v
            matrix v = v[17...,17...]
            matrix se = vecdiag(cholesky(diag(vecdiag(v))))'
            matrix list se
            svmat se, names(se4)
            clist se4 in 1/48

            gen ll4 = est4 - 1.96*se4
            gen ul4 = est4 + 1.96*se4
            clist est4-ul4 in 1/49
```

```
gen marital_statusF = int((mod(_n-1,48))/16)+1 in 1/48
replace marital_statusF = marital_statusF + 1
gen genderF2 = int((mod(_n-1,16))/8)+1 in 1/48
gen proximity_to_deathF2 = int((mod(_n-1,8))/1)+1 in 1/48


est restore maritalstatus2
margins, dydx(marital_status) at(age_at_death = 0 gender = (1 2) proximity_to_death = (1 2 3 4 5 6 7 8)) post

matrix b = e(b)'
matrix list b
matrix b = b[17...,1]
svmat b, names(est5)

matrix v = e(V)
matrix list v
matrix v = v[17...,17...]
matrix se = vecdiag(cholesky(diag(vecdiag(v))))'
matrix list se
svmat se, names(se5)
clist se5 in 1/49

gen ll5 = est5 - 1.96*se5
gen ul5 = est5 + 1.96*se5
clist est5-ul5 in 1/49


est restore maritalstatus3
margins, dydx(marital_status) at(age_at_death = 0 gender = (1 2) proximity_to_death = (1 2 3 4 5 6 7 8)) post

matrix b = e(b)'
matrix list b
matrix b = b[17...,1]
svmat b, names(est6)

matrix v = e(V)
matrix list v
matrix v = v[17...,17...]
matrix se = vecdiag(cholesky(diag(vecdiag(v))))'
matrix list se
svmat se, names(se6)
clist se6 in 1/49

gen ll6 = est6 - 1.96*se6
gen ul6 = est6 + 1.96*se6
clist est6-ul6 in 1/49
```

```
* Graph

            graph twoway ///
            (line yline proximity_to_deathF2, lpattern(solid) lcolor(gs0)) ///
            (line est4 proximity_to_deathF2 if marital_statusF == 2 & genderF2 == 1, lpattern(solid) lcolor(gs12) ) ///
            (line est4 proximity_to_deathF2 if marital_statusF == 3 & genderF2 == 1, lpattern(longdash_dot) lcolor(gs8)) ///
            (line est4 proximity_to_deathF2 if marital_statusF == 4 & genderF2 == 1, lpattern(dash) lcolor(gs4)) ///
              (rcap ll4 ul4 proximity_to_deathF if marital_statusF == 2 & genderF2 == 1, lpattern(solid) lcolor(gs12) lwidth(thin))  ///
              (rcap ll4 ul4 proximity_to_deathF if marital_statusF == 3 & genderF2 == 1, lpattern(solid) lcolor(gs8) lwidth(thin)) ///
              (rcap ll4 ul4 proximity_to_deathF if marital_statusF == 4 & genderF2 == 1, lpattern(solid) lcolor(gs4) lwidth(thin)),  ///
                        xtitle("Years before death", size(large)) ///
                        xlabel(0.5 "8"  1.5 "7"  2.5 "6" 3.5 "5"  4.5 "4"  5.5 "3"  6.5 "2" 7.5 "1" 8.5 "0", labs(large) ) ///
                        ytitle("Dementia" " " "â^†", size(large)) ///
                        ylabel( 0(0.2)0.4, labs(large))     ///
                        legend(order(1 "Married (ref.)" 2 "Divorced" 3 "Widowed" 4 "Never married") size(large) textfirst cols(4) symxsize(6)
span region(col(white))) ///
                        graphr(color(white)) ///
                        subtitle("Men", size(large)) ///
                        aspect(0.5) ///
                        name(fig3_1, replace)

            graph twoway ///
            (line yline proximity_to_deathF2, lpattern(solid) lcolor(gs0)) ///
            (line est4 proximity_to_deathF2 if marital_statusF == 2 & genderF2 == 2, lpattern(solid) lcolor(gs12) ) ///
            (line est4 proximity_to_deathF2 if marital_statusF == 3 & genderF2 == 2, lpattern(longdash_dot) lcolor(gs8)) ///
            (line est4 proximity_to_deathF2 if marital_statusF == 4 & genderF2 == 2, lpattern(dash) lcolor(gs4)) ///
              (rcap ll4 ul4 proximity_to_deathF if marital_statusF == 2 & genderF2 == 2, lpattern(solid) lcolor(gs12) lwidth(thin))  ///
              (rcap ll4 ul4 proximity_to_deathF if marital_statusF == 3 & genderF2 == 2, lpattern(solid) lcolor(gs8) lwidth(thin)) ///
              (rcap ll4 ul4 proximity_to_deathF if marital_statusF == 4 & genderF2 == 2, lpattern(solid) lcolor(gs4) lwidth(thin)),  ///
                        xtitle("Years before death", size(large)) ///
                        xlabel(0.5 "8"  1.5 "7"  2.5 "6" 3.5 "5"  4.5 "4"  5.5 "3"  6.5 "2" 7.5 "1" 8.5 "0", labs(large) ) ///
                        ytitle(" " " " "â^†", size(large)) ///
                        ylabel( 0(0.2)0.4, labs(large))     ///
                        graphr(color(white)) ///
                        legend(order(1 "Married (ref.)" 2 "Divorced" 3 "Widowed" 4 "Never married") size(large) textfirst cols(4) symxsize(6)
span region(col(white))) ///
                        subtitle("Women", size(large)) ///
                        aspect(0.5) ///
                        name(fig3_2, replace)

            graph twoway ///
            (line yline proximity_to_deathF2, lpattern(solid) lcolor(gs0)) ///
            (line est5 proximity_to_deathF2 if marital_statusF == 2 & genderF2 == 1, lpattern(solid) lcolor(gs12) ) ///
            (line est5 proximity_to_deathF2 if marital_statusF == 3 & genderF2 == 1, lpattern(longdash_dot) lcolor(gs8)) ///
            (line est5 proximity_to_deathF2 if marital_statusF == 4 & genderF2 == 1, lpattern(dash) lcolor(gs4)) ///
              (rcap ll5 ul5 proximity_to_deathF if marital_statusF == 2 & genderF2 == 1, lpattern(solid) lcolor(gs12) lwidth(thin))  ///
```

```
          (rcap ll5 ul5 proximity_to_deathF if marital_statusF == 3 & genderF2 == 1, lpattern(solid) lcolor(gs8) lwidth(thin)) ///
          (rcap ll5 ul5 proximity_to_deathF if marital_statusF == 4 & genderF2 == 1, lpattern(solid) lcolor(gs4) lwidth(thin)),  ///
               xtitle("Years before death", size(large)) ///
               xlabel(0.5 "8"  1.5 "7"  2.5 "6" 3.5 "5"  4.5 "4"  5.5 "3"  6.5 "2" 7.5 "1" 8.5 "0", labs(large) ) ///
               ytitle("Non-dementia" " " "â^†", size(large)) ///
               ylabel( 0(0.2)0.4, labs(large))     ///
               graphr(color(white)) ///
               legend(order(1 "Married (ref.)" 2 "Divorced" 3 "Widowed" 4 "Never married") size(large) textfirst cols(4) symxsize(6)
span region(col(white))) ///
               subtitle(" ", size(large)) ///
               aspect(0.5) ///
               name(fig3_3, replace)

        graph twoway ///
        (line yline proximity_to_deathF2, lpattern(solid) lcolor(gs0)) ///
        (line est5 proximity_to_deathF2 if marital_statusF == 2 & genderF2 == 2, lpattern(solid) lcolor(gs12) ) ///
        (line est5 proximity_to_deathF2 if marital_statusF == 3 & genderF2 == 2, lpattern(longdash_dot) lcolor(gs8)) ///
        (line est5 proximity_to_deathF2 if marital_statusF == 4 & genderF2 == 2, lpattern(dash) lcolor(gs4)) ///
          (rcap ll5 ul5 proximity_to_deathF if marital_statusF == 2 & genderF2 == 2, lpattern(solid) lcolor(gs12) lwidth(thin))  ///
          (rcap ll5 ul5 proximity_to_deathF if marital_statusF == 3 & genderF2 == 2, lpattern(solid) lcolor(gs8) lwidth(thin)) ///
          (rcap ll5 ul5 proximity_to_deathF if marital_statusF == 4 & genderF2 == 2, lpattern(solid) lcolor(gs4) lwidth(thin)),  ///
               xtitle("Years before death", size(large))
        ///
               xlabel(0.5 "8"  1.5 "7"  2.5 "6" 3.5 "5"  4.5 "4"  5.5 "3"  6.5 "2" 7.5 "1" 8.5 "0", labs(large) )          ///
               ytitle(" " " " "â^†", size(large)) ///
               ylabel( 0(0.2)0.4, labs(large))
               ///
               graphr(color(white))
                           ///
               legend(order(1 "Married (ref.)" 2 "Divorced" 3 "Widowed" 4 "Never married") size(large) textfirst cols(4) symxsize(6)
span region(col(white))) ///
               subtitle(" ", size(large)) ///
               aspect(0.5) ///
               name(fig3_4, replace)

        graph twoway ///
        (line yline proximity_to_deathF2, lpattern(solid) lcolor(gs0)) ///
        (line est6 proximity_to_deathF2 if marital_statusF == 2 & genderF2 == 1, lpattern(solid) lcolor(gs12) ) ///
        (line est6 proximity_to_deathF2 if marital_statusF == 3 & genderF2 == 1, lpattern(longdash_dot) lcolor(gs8)) ///
        (line est6 proximity_to_deathF2 if marital_statusF == 4 & genderF2 == 1, lpattern(dash) lcolor(gs4)) ///
          (rcap ll6 ul6 proximity_to_deathF if marital_statusF == 2 & genderF2 == 1, lpattern(solid) lcolor(gs12) lwidth(thin))  ///
          (rcap ll6 ul6 proximity_to_deathF if marital_statusF == 3 & genderF2 == 1, lpattern(solid) lcolor(gs8) lwidth(thin)) ///
          (rcap ll6 ul6 proximity_to_deathF if marital_statusF == 4 & genderF2 == 1, lpattern(solid) lcolor(gs4) lwidth(thin)),  ///
               xtitle("Years before the end of the study period", size(large)) ///
               xlabel(0.5 "8"  1.5 "7"  2.5 "6" 3.5 "5"  4.5 "4"  5.5 "3"  6.5 "2" 7.5 "1" 8.5 "0", labs(large) ) ///
               ytitle("Survived" " " "â^†", size(large)) ///
               ylabel( 0(0.2)0.4, labs(large))     ///
               graphr(color(white)) ///
```

```
                        legend(order(1 "Married (ref.)" 2 "Divorced" 3 "Widowed" 4 "Never married") size(large) textfirst cols(4) symxsize(6)
span region(col(white))) ///
                        subtitle(" ", size(large)) ///
                        aspect(0.5) ///
                        name(fig3_5, replace)


            graph twoway ///
            (line yline proximity_to_deathF2, lpattern(solid) lcolor(gs0)) ///
            (line est6 proximity_to_deathF2 if marital_statusF == 2 & genderF2 == 2, lpattern(solid) lcolor(gs12) ) ///
            (line est6 proximity_to_deathF2 if marital_statusF == 3 & genderF2 == 2, lpattern(longdash_dot) lcolor(gs8)) ///
            (line est6 proximity_to_deathF2 if marital_statusF == 4 & genderF2 == 2, lpattern(dash) lcolor(gs4)) ///
              (rcap ll6 ul6 proximity_to_deathF if marital_statusF == 2 & genderF2 == 2, lpattern(solid) lcolor(gs12) lwidth(thin))  ///
              (rcap ll6 ul6 proximity_to_deathF if marital_statusF == 3 & genderF2 == 2, lpattern(solid) lcolor(gs8) lwidth(thin)) ///
              (rcap ll6 ul6 proximity_to_deathF if marital_statusF == 4 & genderF2 == 2, lpattern(solid) lcolor(gs4) lwidth(thin)),  ///
                        xtitle("Years before the end of the study period", size(large)) ///
                        xlabel(0.5 "8"  1.5 "7"  2.5 "6" 3.5 "5"  4.5 "4"  5.5 "3"  6.5 "2" 7.5 "1" 8.5 "0", labs(large) ) ///
                        ytitle(" " " " "â ̂†", size(large)) ///
                        ylabel( 0(0.2)0.4, labs(large))     ///
                        graphr(color(white)) ///
                        legend(order(1 "Married (ref.)" 2 "Divorced" 3 "Widowed" 4 "Never married") size(large) textfirst cols(4) symxsize(6)
span region(col(white))) ///
                        subtitle(" ", size(large)) ///
                        aspect(0.5) ///
                        name(fig3_6, replace)



/*************************************************************************************************************
Figure 4: Household income differences in the probability (%) of institutional long-term care (LTC) according to proximity
to death (or the and of the study period for survivors) and by gender and cause of death; Finnish men and women,
1995-2007
************************************************************************************************************/

* Fit the models and save results

            xtgee ltc c.calendar_year i.region_of_residence c.age_at_death c.age_at_death2 i.gender i.marital_status i.household_income
c.proximity_to_death c.proximity_to_death2 c.proximity_to_death3 c.proximity_to_death#i.household_income#i.gender if cause_of_death == 1
[pweight=npaino], ///
              eform fam(binomial) link(logit) i(id) t(proximity_to_death) corr(ar1)
            est store householdincome1

            xtgee ltc c.calendar_year i.region_of_residence c.age_at_death c.age_at_death2 i.gender i.marital_status i.household_income
c.proximity_to_death c.proximity_to_death2 c.proximity_to_death3 c.proximity_to_death#i.household_income#i.gender if cause_of_death == 2
[pweight=npaino], ///
              eform fam(binomial) link(logit) i(id) t(proximity_to_death) corr(ar1)
            est store householdincome2
```

```
            xtgee ltc c.calendar_year i.region_of_residence c.age_at_death c.age_at_death2 i.gender i.marital_status i.household_income
c.proximity_to_death c.proximity_to_death2 c.proximity_to_death3 c.proximity_to_death#i.household_income#i.gender if cause_of_death == 3
[pweight=npaino], ///
              eform fam(binomial) link(logit) i(id) t(proximity_to_death) corr(ar1)
            est store householdincome3


* Estimate marginal effects

            est restore householdincome1
            margins, dydx(household_income) at(gender = (1 2) proximity_to_death = (1 2 3 4 5 6 7 8)) post

            matrix b = e(b)'
            matrix list b
            matrix b = b[17...,1]
            svmat b, names(est7)

            matrix v = e(V)
            matrix list v
            matrix v = v[17...,17...]
            matrix se = vecdiag(cholesky(diag(vecdiag(v))))'
            matrix list se
            svmat se, names(se7)
            clist se7 in 1/64

            gen ll7 = est7 - 1.96*se7
            gen ul7 = est7 + 1.96*se7
            clist est7-ul7 in 1/65

            gen household_incomeF = int((mod(_n-1,64))/16)+1 in 1/64
            replace household_incomeF = household_incomeF + 1


            est restore householdincome2
            margins, dydx(household_income) at(gender = (1 2) proximity_to_death = (1 2 3 4 5 6 7 8)) post

            matrix b = e(b)'
            matrix list b
            matrix b = b[17...,1]
            svmat b, names(est8)

            matrix v = e(V)
            matrix list v
            matrix v = v[17...,17...]
            matrix se = vecdiag(cholesky(diag(vecdiag(v))))'
            matrix list se
            svmat se, names(se8)
            clist se8 in 1/64
```

```
            gen ll8 = est8 - 1.96*se8
            gen ul8 = est8 + 1.96*se8
            clist est8-ul8 in 1/65


            est restore householdincome3
            margins, dydx(household_income) at(gender = (1 2) proximity_to_death = (1 2 3 4 5 6 7 8)) post

            matrix b = e(b)'
            matrix list b
            matrix b = b[17...,1]
            svmat b, names(est9)

            matrix v = e(V)
            matrix list v
            matrix v = v[17...,17...]
            matrix se = vecdiag(cholesky(diag(vecdiag(v))))'
            matrix list se
            svmat se, names(se9)
            clist se9 in 1/64

            gen ll9 = est9 - 1.96*se9
            gen ul9 = est9 + 1.96*se9
            clist est9-ul9 in 1/65


* Graph

            graph twoway ///
            (line yline proximity_to_deathF, lpattern(solid) lcolor(gs0)) ///
            (line est7 proximity_to_deathF if household_incomeF == 2 & genderF == 1, lpattern(solid) lcolor(gs12) )      ///
            (line est7 proximity_to_deathF if household_incomeF == 3 & genderF == 1, lpattern(longdash_dot) lcolor(gs8)) ///
            (line est7 proximity_to_deathF if household_incomeF == 4 & genderF == 1, lpattern(dash) lcolor(gs4)) ///
            (line est7 proximity_to_deathF if household_incomeF == 5 & genderF == 1, lpattern(shortdash_dot) lcolor(gs0)) ///

              (rcap ll7 ul7 proximity_to_deathF if household_incomeF == 2 & genderF == 1, lpattern(solid) lcolor(gs12) lwidth(thin)) ///
              (rcap ll7 ul7 proximity_to_deathF if household_incomeF == 3 & genderF == 1, lpattern(solid) lcolor(gs8) lwidth(thin))  ///
              (rcap ll7 ul7 proximity_to_deathF if household_incomeF == 4 & genderF == 1, lpattern(solid) lcolor(gs4) lwidth(thin))  ///
              (rcap ll7 ul7 proximity_to_deathF if household_incomeF == 5 & genderF == 1, lpattern(solid) lcolor(gs0) lwidth(thin)), ///
                        xtitle("Years before death", size(large)) ///
                        xlabel(0.5 "8"  1.5 "7"  2.5 "6" 3.5 "5"  4.5 "4"  5.5 "3"  6.5 "2" 7.5 "1" 8.5 "0", labs(large) ) ///
                        ytitle("Dementia" " " "â^†", size(large)) ///
                        ylabel( 0(0.2)0.4, labs(large))     ///
                        graphr(color(white)) ///
                        legend(order(1 "Highest (ref.)" 2 "2nd" 3 "3rd" 4 "4th" 5 "Lowest") size(large) textfirst cols(5) symxsize(6) span
region(col(white))) ///
                        subtitle("Men", size(large)) ///
```

```
                    aspect(0.5) ///
                    name(fig4_1, replace)

        graph twoway ///
        (line yline proximity_to_deathF, lpattern(solid) lcolor(gs0)) ///
        (line est7 proximity_to_deathF if household_incomeF == 2 & genderF == 2, lpattern(solid) lcolor(gs12) )      ///
        (line est7 proximity_to_deathF if household_incomeF == 3 & genderF == 2, lpattern(longdash_dot) lcolor(gs8)) ///
        (line est7 proximity_to_deathF if household_incomeF == 4 & genderF == 2, lpattern(dash) lcolor(gs4)) ///
        (line est7 proximity_to_deathF if household_incomeF == 5 & genderF == 2, lpattern(shortdash_dot) lcolor(gs0)) ///

          (rcap ll7 ul7 proximity_to_deathF if household_incomeF == 2 & genderF == 2, lpattern(solid) lcolor(gs12) lwidth(thin)) ///
          (rcap ll7 ul7 proximity_to_deathF if household_incomeF == 3 & genderF == 2, lpattern(solid) lcolor(gs8) lwidth(thin))  ///
          (rcap ll7 ul7 proximity_to_deathF if household_incomeF == 4 & genderF == 2, lpattern(solid) lcolor(gs4) lwidth(thin))  ///
          (rcap ll7 ul7 proximity_to_deathF if household_incomeF == 5 & genderF == 2, lpattern(solid) lcolor(gs0) lwidth(thin)), ///
                    xtitle("Years before death", size(large)) ///
                    xlabel(0.5 "8"  1.5 "7"  2.5 "6" 3.5 "5"  4.5 "4"  5.5 "3"  6.5 "2" 7.5 "1" 8.5 "0", labs(large) ) ///
                    ytitle(" " " " "âˆ†", size(large)) ///
                    ylabel( 0(0.2)0.4, labs(large))     ///
                    yscale(range(-0.02/0.4)) ///
                    graphr(color(white)) ///
                    legend(order(1 "Highest (ref.)" 2 "2nd" 3 "3rd" 4 "4th" 5 "Lowest") size(large) textfirst cols(5) symxsize(6) span
region(col(white))) ///
                    subtitle("Women", size(large)) ///
                    aspect(0.5) ///
                    name(fig4_2, replace)

        graph twoway ///
        (line yline proximity_to_deathF, lpattern(solid) lcolor(gs0)) ///
        (line est8 proximity_to_deathF if household_incomeF == 2 & genderF == 1, lpattern(solid) lcolor(gs12) )      ///
        (line est8 proximity_to_deathF if household_incomeF == 3 & genderF == 1, lpattern(longdash_dot) lcolor(gs8)) ///
        (line est8 proximity_to_deathF if household_incomeF == 4 & genderF == 1, lpattern(dash) lcolor(gs4)) ///
        (line est8 proximity_to_deathF if household_incomeF == 5 & genderF == 1, lpattern(shortdash_dot) lcolor(gs0)) ///

          (rcap ll8 ul8 proximity_to_deathF if household_incomeF == 2 & genderF == 1, lpattern(solid) lcolor(gs12) lwidth(thin)) ///
          (rcap ll8 ul8 proximity_to_deathF if household_incomeF == 3 & genderF == 1, lpattern(solid) lcolor(gs8) lwidth(thin))  ///
          (rcap ll8 ul8 proximity_to_deathF if household_incomeF == 4 & genderF == 1, lpattern(solid) lcolor(gs4) lwidth(thin))  ///
          (rcap ll8 ul8 proximity_to_deathF if household_incomeF == 5 & genderF == 1, lpattern(solid) lcolor(gs0) lwidth(thin)), ///
                    xtitle("Years before death", size(large)) ///
                    xlabel(0.5 "8"  1.5 "7"  2.5 "6" 3.5 "5"  4.5 "4"  5.5 "3"  6.5 "2" 7.5 "1" 8.5 "0", labs(large) ) ///
                    ytitle("Non-dementia" " " "âˆ†", size(large)) ///
                    ylabel( 0(0.2)0.4, labs(large))     ///
                    yscale(range(-0.02/0.4)) ///
                    graphr(color(white)) ///
                    legend(order(1 "Highest (ref.)" 2 "2nd" 3 "3rd" 4 "4th" 5 "Lowest") size(large) textfirst cols(5) symxsize(6) span
region(col(white))) ///
                    subtitle(" ", size(large)) ///
                    aspect(0.5) ///
                    name(fig4_3, replace)
```

```
            graph twoway ///
            (line yline proximity_to_deathF, lpattern(solid) lcolor(gs0)) ///
            (line est8 proximity_to_deathF if household_incomeF == 2 & genderF == 2, lpattern(solid) lcolor(gs12) )      ///
            (line est8 proximity_to_deathF if household_incomeF == 3 & genderF == 2, lpattern(longdash_dot) lcolor(gs8)) ///
            (line est8 proximity_to_deathF if household_incomeF == 4 & genderF == 2, lpattern(dash) lcolor(gs4)) ///
            (line est8 proximity_to_deathF if household_incomeF == 5 & genderF == 2, lpattern(shortdash_dot) lcolor(gs0)) ///

              (rcap ll8 ul8 proximity_to_deathF if household_incomeF == 2 & genderF == 2, lpattern(solid) lcolor(gs12) lwidth(thin)) ///
              (rcap ll8 ul8 proximity_to_deathF if household_incomeF == 3 & genderF == 2, lpattern(solid) lcolor(gs8) lwidth(thin))  ///
              (rcap ll8 ul8 proximity_to_deathF if household_incomeF == 4 & genderF == 2, lpattern(solid) lcolor(gs4) lwidth(thin))  ///
              (rcap ll8 ul8 proximity_to_deathF if household_incomeF == 5 & genderF == 2, lpattern(solid) lcolor(gs0) lwidth(thin)), ///
                        xtitle("Years before death", size(large)) ///
                        xlabel(0.5 "8"  1.5 "7"  2.5 "6" 3.5 "5"  4.5 "4"  5.5 "3"  6.5 "2" 7.5 "1" 8.5 "0", labs(large) ) ///
                        ytitle(" " " " "â^†", size(large)) ///
                        ylabel( 0(0.2)0.4, labs(large))     ///
                        yscale(range(-0.02/0.4)) ///
                        graphr(color(white)) ///
                        legend(order(1 "Highest (ref.)" 2 "2nd" 3 "3rd" 4 "4th" 5 "Lowest") size(large) textfirst cols(5) symxsize(6) span
region(col(white))) ///
                        subtitle(" ", size(large)) ///
                        aspect(0.5) ///
                        name(fig4_4, replace)

            graph twoway ///
            (line yline proximity_to_deathF, lpattern(solid) lcolor(gs0)) ///
            (line est9 proximity_to_deathF if household_incomeF == 2 & genderF == 1, lpattern(solid) lcolor(gs12) )      ///
            (line est9 proximity_to_deathF if household_incomeF == 3 & genderF == 1, lpattern(longdash_dot) lcolor(gs8)) ///
            (line est9 proximity_to_deathF if household_incomeF == 4 & genderF == 1, lpattern(dash) lcolor(gs4)) ///
            (line est9 proximity_to_deathF if household_incomeF == 5 & genderF == 1, lpattern(shortdash_dot) lcolor(gs0)) ///

              (rcap ll9 ul9 proximity_to_deathF if household_incomeF == 2 & genderF == 1, lpattern(solid) lcolor(gs12) lwidth(thin)) ///
              (rcap ll9 ul9 proximity_to_deathF if household_incomeF == 3 & genderF == 1, lpattern(solid) lcolor(gs8) lwidth(thin))  ///
              (rcap ll9 ul9 proximity_to_deathF if household_incomeF == 4 & genderF == 1, lpattern(solid) lcolor(gs4) lwidth(thin))  ///
              (rcap ll9 ul9 proximity_to_deathF if household_incomeF == 5 & genderF == 1, lpattern(solid) lcolor(gs0) lwidth(thin)), ///
                        xtitle("Years before the end of the study period", size(large))         ///
                        xlabel(0.5 "8"  1.5 "7"  2.5 "6" 3.5 "5"  4.5 "4"  5.5 "3"  6.5 "2" 7.5 "1" 8.5 "0", labs(large) ) ///
                        ytitle("Survived" " " "â^†", size(large)) ///
                        ylabel( 0(0.2)0.4, labs(large))     ///
                        yscale(range(-0.02/0.4)) ///
                        graphr(color(white)) ///
                        legend(order(1 "Highest (ref.)" 2 "2nd" 3 "3rd" 4 "4th" 5 "Lowest") size(large) textfirst cols(5) symxsize(6) span
region(col(white))) ///
                        subtitle(" ", size(large)) ///
                        aspect(0.5) ///
                        name(fig4_5, replace)

            graph twoway ///
```

```
          (line yline proximity_to_deathF, lpattern(solid) lcolor(gs0)) ///
          (line est9 proximity_to_deathF if household_incomeF == 2 & genderF == 2, lpattern(solid) lcolor(gs12) )      ///
          (line est9 proximity_to_deathF if household_incomeF == 3 & genderF == 2, lpattern(longdash_dot) lcolor(gs8)) ///
          (line est9 proximity_to_deathF if household_incomeF == 4 & genderF == 2, lpattern(dash) lcolor(gs4)) ///
          (line est9 proximity_to_deathF if household_incomeF == 5 & genderF == 2, lpattern(shortdash_dot) lcolor(gs0)) ///

            (rcap ll9 ul9 proximity_to_deathF if household_incomeF == 2 & genderF == 2, lpattern(solid) lcolor(gs12) lwidth(thin)) ///
            (rcap ll9 ul9 proximity_to_deathF if household_incomeF == 3 & genderF == 2, lpattern(solid) lcolor(gs8) lwidth(thin))  ///
            (rcap ll9 ul9 proximity_to_deathF if household_incomeF == 4 & genderF == 2, lpattern(solid) lcolor(gs4) lwidth(thin))  ///
            (rcap ll9 ul9 proximity_to_deathF if household_incomeF == 5 & genderF == 2, lpattern(solid) lcolor(gs0) lwidth(thin)), ///
                        xtitle("Years before the end of the study period", size(large))          ///
                        xlabel(0.5 "8"  1.5 "7"  2.5 "6" 3.5 "5"  4.5 "4"  5.5 "3"  6.5 "2" 7.5 "1" 8.5 "0", labs(large) ) ///
                        ytitle(" " " " "â^†", size(large)) ///
                        ylabel( 0(0.2)0.4, labs(large))     ///
                        yscale(range(-0.02/0.4)) ///
                        graphr(color(white)) ///
                        legend(order(1 "Highest (ref.)" 2 "2nd" 3 "3rd" 4 "4th" 5 "Lowest") size(large) textfirst cols(5) symxsize(6) span
region(col(white))) ///
                        subtitle(" ", size(large)) ///
                        aspect(0.5) ///
                        name(fig4_6, replace)
```
